# Linking primary emotional traits to ideological attitudes and personal value types

**DOI:** 10.1371/journal.pone.0279885

**Published:** 2023-01-03

**Authors:** Cornelia Sindermann, Christopher Kannen, Christian Montag

**Affiliations:** 1 Department of Molecular Psychology, Institute of Psychology and Education, Ulm University, Ulm, Germany; 2 Department of Psychology, University of Toledo, Toledo, OH, United States of America; Universitat de Valencia, SPAIN

## Abstract

The present study aimed at investigating associations of both ideological attitudes and personal value types with the personality traits derived from the Affective Neuroscience Theory (ANT). For that, data of N = 626 (n = 403 men, n = 220 women, n = 3 identifying as neither a man nor a woman) participants of an online survey in the German language were analyzed. Relations of primary emotional traits derived from the ANT with Right-Wing Authoritarianism (RWA), Social Dominance Orientation (SDO), and personal value types, such as the higher-order value type dimensions “Conservation–Openness to Change” and “Self-Enhancement–Self-Transcendence”, were examined by means of correlational analyses and structural equation modeling. Results revealed among others relations between low SEEKING, high ANGER and high RWA. Low CARE and high ANGER were associated with high SDO. Moreover, FEAR was related to the higher-order value type dimension ranging from Conservation to Openness to Change. ANGER was associated with the higher-order value type dimension ranging from Self-Enhancement to Self-Transcendence. The present results do not only expand knowledge on the personality traits associated with ideological attitudes and personal value types. Beyond this, considering the neuroanatomical, functional, and neurochemical correlates of the primary emotional traits SEEKING, ANGER, CARE, and FEAR, the present results may provide a roadmap for forthcoming studies aiming at examining biological correlates of ideological attitudes and personal value types, such as those works in the field of political neuroscience.

## Introduction

Understanding citizens’ political views is an important aim not only for researchers in the fields of psychology and political sciences. Next to environmental factors, also dispositional characteristics have been assumed to be associated with individual differences in political views (e.g., for authoritarianism [1]). Such dispositional factors include personality traits and indeed, various studies found (small) associations between individuals’ personality traits and various measures of political views [2–5].

The present study sought to contribute to the research on examining dispositional characteristics associated with individual differences in political views. More specifically, it was aimed at investigating the links of personality traits derived from the Affective Neuroscience Theory (ANT) with the two ideological attitudes Social Dominance Orientation (SDO) and Right-Wing Authoritarianism (RWA) of the Dual-Process Motivational Model [6–8], as well as with personal value types being related to political views [9,10]. The personality traits derived from the ANT have not yet been investigated in light of ideological attitudes and value types. Thus, this work expands knowledge in the research field on the personality trait associations of political views. As shortly discussed later in the present work, given the biological approach of the ANT, the present results may additionally provide a roadmap for further investigations on specific brain structures and functions, as well as neurotransmitters/-peptides putatively being related to RWA, SDO, and value types. Hence, the present findings may build a bridge between the research fields of personality psychology and political neuroscience.

### Personality traits derived from the Affective Neuroscience Theory

The present work focuses on the so-called Primary Emotional Traits (PETs) SEEKING, FEAR, CARE, ANGER, PLAY, and SADNESS derived from the ANT. These terms are capitalized based on the nomenclature put forward in the ANT by Jaak Panksepp in order to ensure a specialized terminology for those experiential processes and their biological roots (see Discussion section). The capitalization also supports a clear distinction of PETs from other psychological constructs labeled alike but actually constituting different concepts according to Panksepp [11–13]. In the present work, the capitalization of the PETs is used in line with the convention do so (see nearly all publications on the PETs by various authors). Contrary to the lexical approach resulting, for instance, in the prominent Five-Factor Model and the Big Five of personality [14], the PETs were built upon the ANT and abundant neuroscientific research in mammals [11,15,16]; for an overview on Pankseppian principles in Affective Neuroscience see Panksepp [16]. In humans, high scores in the PET SEEKING are related to enjoying problem-solving, making new experiences, exploring new areas, and being curious. High scores in FEAR describe individuals who are anxious, troubled, generally more afraid, and worry a lot. High scores in CARE are linked to taking care of other humans and animals. High ANGER is associated with getting angry and hotheaded rather easily, which can be triggered by frustration or when protecting one’s resources. High scores in PLAY describe individuals enjoying fun and playing games with others. Finally, high SADNESS is related to feeling lonely, being sad and crying often [11].

Various researchers have investigated the ANT, and PETs specifically, in humans by applying a personality trait approach and the so-called Affective Neuroscience Personality Scales (ANPS) in various languages [17–19]. One theoretical study has linked PETs to emotions like those based on Ekman’s facial expressions (e.g., SEEKING to Joy, FEAR to fear, ANGER to anger, SADNESS to sadness [20]). In addition, PETs have been associated with trait characteristics like the Big Five of personality in numerous studies [21]. Especially three of the Big Five traits, and the PETs being related to them, are of considerable interest for the present work: Openness, Agreeableness, and Conscientiousness. High scores in Openness describe individuals who are open to new ideas and art, like to make new experiences, and/or like to get to know, for example, other cultures. Highly agreeable individuals are altruistic, compliant, and/or cooperative. Individuals scoring high in Conscientiousness are orderly, carry out their duties, and/or are self-disciplined [22–24]. SEEKING and Openness are strongly positively related. Agreeableness is most strongly positively related to CARE and most strongly negatively associated with ANGER. Other relations of Openness and Agreeableness with PETs exist but are smaller than the previously mentioned ones. Finally, Conscientiousness does not seem to be consistently/strongly associated with any of the PETs, which is not surprising given that this trait might be the most “cognitively controlled” one of the Big Five [21].

### The Dual-Process Motivational Model–Right-Wing Authoritarianism and social dominance orientation

The Dual-Process Motivational Model by Duckitt and Sibley [7] constitutes two positively related but distinct ideological attitudes, namely RWA and SDO, being associated with socio-political and intergroup behaviors and outcomes. RWA comprises authoritarian submission, authoritarian aggression, and conventionalism [25]. SDO describes to what extent individuals tend to favor hierarchies between social groups (high SDO: favoring hierarchies; Pratto et al. [26]). Two different world views and different personality traits are considered to be linked to each ideological attitude according to the Dual-Process Motivational Model. On the one hand, the model constitutes low Openness and high Conscientiousness being related to “dangerous world” beliefs, which in turn are associated with higher RWA. On the other hand, the model constitutes low Agreeableness to be related to the “competitive-jungle” worldview, which is in turn associated with higher SDO [7].

Related to direct and bivariate associations between personality traits and ideological attitudes and mostly in line with the theoretical model, empirical research shows significant positive associations of RWA with Conscientiousness across studies [27–31]. In most but not all of these studies, also a negative association of RWA with Openness is reported. A meta-analysis supports findings on a positive association of Conscientiousness and a negative association of Openness with RWA [32]. Moreover, across many studies, SDO has been negatively related to Agreeableness [27,29–31]. A meta-analysis concludes that next to Agreeableness, also Openness is negatively related to SDO [32].

Based on the model by Duckitt and Sibley [7], the relations of RWA and SDO with certain Big Five traits (e.g., Openness, Agreeableness), and the associations of the same Big Five traits with certain PETs described in detail before, the following hypotheses can be stated [H2 was not included in the preregistration but added later based on relations of SEEKING, RWA, and SDO with Openness reported above]:

- H1: RWA and SDO are positively related.
- H2: SEEKING is negatively related to both RWA and SDO.
- H3: CARE is negatively related to SDO.
- H4: ANGER is positively related to SDO.

Taking into account the two worldviews related to each RWA (world as dangerous place) and SDO (world as competitive jungle), strengthens the idea of SDO being positively related to ANGER (being related to protecting one’s own resources) and additionally suggests the following hypothesis:

- H5: FEAR is positively related to RWA.

H5 is also supported by literature showing moderate positive relations between RWA and emotions of being fearful, threatened, anxious, and concerned by various life events [33].

### Personal value types according to Schwartz

According to Shalom Schwartz and his theory of basic human values [9], every human holds ten different value types to various degrees. These value types are labeled Self-Direction, Stimulation, Hedonism, Achievement, Power, Security, Conformity, Tradition, Benevolence, and Universalism. According to the theory, they are ordered in a circumplex model. Value types close to each other in the model are thought to be positively related and value types on opposite sides of the model negatively. More specifically, the value types of Self-Direction, Stimulation (and Hedonism) contribute to the higher order value type “Openness to Change” according to the theory. This higher-order value type lies on the opposite side in the circumplex model as does the higher-order “Conservation” value type comprising Security, Conformity, and Tradition. Thus, the two higher order value types Openness to Change and Conservation span a dimension with opposing value types on each side. Similarly, the value types Benevolence and Universalism are collapsed in the higher-order value type “Self-Transcendence”, which is opposing the higher-order value type “Self-Enhancement” comprising the value types Achievement and Power (and Hedonism).

In his early work, Schwartz [34] aimed at examining associations between these personal value types and ideologies, attitudes, and behaviors among others in the political field. Additionally, several empirical studies have linked some of the value types to various measures of political views, attitudes, and behaviors in samples from different countries [10,35,36]. Of special interest for the present study, the value types have been linked to the ideological attitudes of RWA and SDO. More specifically, Security, Conformity, and Tradition (building the higher-order Conservation value type) have been positively related to RWA, while Universalism has been negatively related to RWA across various studies [25,37–40]. Next to these relations, some additional significant correlations with other value types were found in some of the cited studies but the previously reported ones are those that were found across all studies. In all cited studies but one [38], Power was positively related to RWA. This is in line with the assumption of Sinn [41] proposing that a positive relation between RWA and Power can be expected. Moreover, in three of four studies in which relations of RWA with Self-Direction and Hedonism were investigated, these relations were significantly negative [37–40].

SDO was found to positively relate to Security (included in the higher-order value type Conservation) as well as to Power and Achievement (building the higher-order value type Self-Enhancement). Moreover, SDO was found to be negatively associated with Universalism and Benevolence across studies (building the higher-order value type Self-Transcendence) [37–39]; additional significant correlations of SDO with other value types were found in some of the studies but reported ones are those that were found across all studies. Sinn [41] additionally mentions a relation between SDO and Hedonism, which was also significantly positive in two of the cited studies [38,39]. The hypotheses on relations of RWA and SDO with value types introduced above are also described in detail in the preregistration of the present work.

Additionally, the value types have been associated with personality traits, among others Openness, Agreeableness, and Conscientiousness, also being related to RWA and SDO: While Openness exhibited positive relations to Self-Direction (strongest relation), Stimulation, and Universalism, it was negatively related to Security, Tradition, and Conformity according to a meta-analysis [42]. Agreeableness was found to be positively associated with Benevolence (strongest association), Universalism, Conformity, and Tradition, and it has been found to negatively relate to Power in the same meta-analysis [42]. Finally, Conscientiousness showed positive correlations with Security (strongest association), Conformity, and Achievement in this meta-analysis [42].

Considering i) the putative relations of SEEKING, CARE, ANGER, and FEAR with RWA and SDO and the relations between RWA and SDO with certain value types, ii) the relations of SEEKING, CARE, and ANGER with certain Big Five traits (e.g., Openness, Agreeableness) and of the same Big Five traits with certain value types, the following hypotheses are postulated [some hypotheses were added after the preregistration based previous research presented before]:

- H6: SEEKING is positively related to Self-Direction, Stimulation, Universalism and Benevolence; it is negatively related to Security, Tradition, and Conformity, as well as Achievement and Power.
- H7: CARE is positively related to Benevolence, Universalism, Conformity, and Tradition; it is negatively related to Security, Power, Achievement, and Hedonism.
- H8: ANGER is positively related to Power, Achievement, Hedonism, and Security; it is negatively related to Benevolence, Universalism, Conformity, and Tradition.

Moreover, since it was expected that FEAR would be related to RWA and RWA seems to be related to several value types, the following hypothesis can be formulated:

- H9: FEAR is positively related to Security, Conformity, Tradition, and Power; it is negatively related to Universalism, Self-Direction, and Hedonism.

Some of the assumed relations in H7 are also supported by relations found between values and the moral foundations scale harm-care [43].

**Fig 1:**
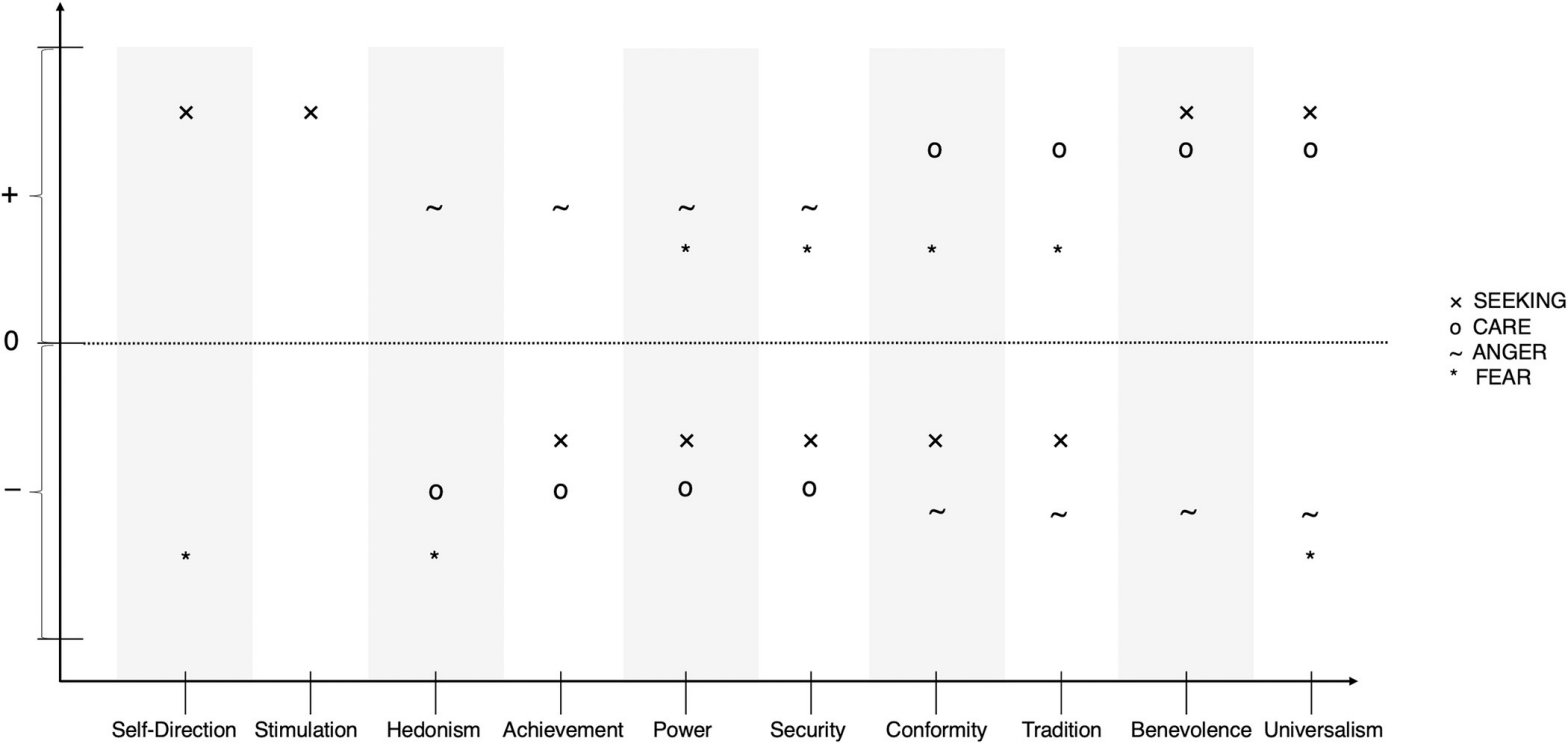
Graphical illustration of the ten value types according to Schwartz’ value theory on the x-axis and expected relations with the primary emotional traits (PETs) of SEEKING, CARE, ANGER, and FEAR on the y-axis. The upper half of the y-axis indicates positive and the lower half negative relations. Different positioning within the upper/lower half does not indicate different strengths of associations; we only formulate hypotheses on the direction of effects (positive versus negative) but not the strengths of effect sizes.

The expected relations of SEEKING, CARE, ANGER, and FEAR with the value types are graphically illustrated in Fig 1.

### Summary of aims of the present study

In summary, the present work aimed at examining the associations of PETs with the ideological attitudes of RWA and SDO as well as with personal value types. By doing so, the knowledge on personality trait associations with ideological attitudes and value types is expanded.

## Materials and methods

### Procedure

The present study including sample size calculations, procedure, measures, and statistical analyses, was preregistered (https://osf.io/bpdg5).

The study was implemented as an online survey on the SurveyCoder platform [44,45]. Data were collected between December 2020 and February 2021. Individuals from Germany eligible to vote in the general German elections in 2021 who were at least 18 years old were allowed to participate. All participants provided informed electronic consent (by clicking on “I agree” after reading detailed participant information, which was provided as PDF for downloading) prior to participation. The online survey was approved by the local ethics committee of Ulm University, Ulm, Germany (nr: 247/20) and the procedure followed the latest revision of the Declaration of Helsinki.

The online survey was advertised offline (printed press, etc.) and online (social media, online magazines, etc.). For example, each time the first author of this work was asked to give an interview on a topic related to the topic of the present survey, the survey link was included in the interview. In addition, Twitter ads were used to distribute information and the link to the survey. Students writing their theses using data from the present survey advertised the study as well. Finally, some online “influencers” also shared advertisements for the survey online.

Given these recruitment strategies, the present sample is a convenience sample. As an incentive, participants received anonymous, pre-programmed feedback on their scores on some of the questionnaires, which they completed during participation. The present sample overlaps with sample 2 in Sindermann et al. [46], in which neither the ANPS nor personal value types were investigated and with data provided in Sindermann et al. [47].

### Sample

After data cleaning (see S1 File), a final sample of N = 626 (n = 403 men, n = 220 women, n = 3 identifying neither as man nor as woman; M(age) = 27.26, SD(age) = 10.71) individuals remained. Most participants (n = 423) indicated no or some kind of school degree as highest educational degree. The remaining n = 203 participants stated university/university of applied sciences degree as their highest educational degree. The final sample size is smaller than the one originally aimed for (N = 779). However, it was not possible to recruit more participants in a reasonable amount of time at reasonable costs. Moreover, the actual final sample size is still pretty large and sufficient to detect small effect sizes in correlational analyses: With a sample size of N = 626, correlations as low as |0.08| can be detected (alpha error probability: 0.05, power = 0.80, two-tailed hypothesis testing, r(H0) = 0.00).

### Measures

#### Personality traits

The German version of the ANPS questionnaire was applied in order to assess individuals’ PET scores [48,49]. A total of 110 items are included in this questionnaire. The six scales of interest for the present work to assess the six PETs comprise 14 items each; the remaining items of the questionnaire are not of interest for the present work. Each item is responded to on a 4-point rating scale from 1 = “strongly disagree” to 4 = “strongly agree”. Cronbach’s alphas for the six scales ranged from 0.74 (SEEKING) to 0.88 (FEAR).

#### Ideological attitudes

The Short Scale on Authoritarianism (KSA-3; abbreviation based on the German name of the scale) [25] was applied to assess RWA. The KSA-3 consists of nine items answered on a 5-point rating scale ranging from 1 = “strongly disagree” to 5 = “strongly agree”. Cronbach’s alpha for the total scale was 0.81. Cronbach’s alphas for the sub-scales with three items each were 0.74 (Authoritarian Aggression), 0.73 (Authoritarian Submission), and 0.67 (Conventionalism) in the present sample.

SDO was assessed by means of the German version (Six, Wolfradt, & Zick (2001) as cited in Mortal [50]) and revised by Sindermann, Schmitt et al. [51]) of the Social Dominance Orientation scale [26]. The SDO scale comprises 16 items, which are answered on a 7-point rating scale from 1 = “very negative” to 7 = “very positive”. Cronbach’s alpha was 0.89 in the present sample.

#### Personal value types

To assess individuals’ scores in personal value types according to Schwartz [9,34], the German version of the 21-item Portraits Value Questionnaire (PVQ) used in the European Social Survey [52] was applied. The items are answered on a 6-point rating scale ranging from 1 = “not like me at all” to 6 = “very much like me”. Inter-item correlations ranged from rho = 0.21 (average inter-item correlation; Universalism) to rho = 0.61 (Stimulation) with the exception of the Tradition scale, which showed an inter-item correlation of -0.07 and was excluded from further analyses, accordingly (note: since all but one scale only comprise two items, no Cronbach’s alphas are presented).

### Statistical analyses

The statistical software R version 4.1.0 [53] and the software R-Studio version 1.4.1106 [54] were used for statistical analyses.

Based on the rule of thumb by Miles and Shevlin [55], a(n approximate) normal distribution could not be assumed for several of the metric scales in the total sample, and the subsamples of men and women, and in the subsamples of individuals with different educational backgrounds. Statistical analyses were adjusted, accordingly.

In more detail, descriptive statistics of all variables of main interest were calculated and associations with age, gender, and educational background were examined by means of Spearman correlations (age was non-normally distributed) and t-tests or Mann-Whitney U-tests. These investigations were of importance to examine whether age, gender, and education needed to be included as control variables in the final analyses.

Next, zero-order (bivariate) correlations of all ANPS scales with the KSA-3 scales, the SDO scale, and all PVQ scales were computed. Afterward, two structural equation models (SEMs) were computed using the lavaan package in R [56]. In the first SEM, both RWA and SDO were modeled as latent factors. While RWA was modeled as a higher-order latent factor with the sub-factors Authoritarian Aggression, Authoritarian Submission, and Conventionalism, SDO was modeled without sub-factors. Both latent factors RWA and SDO were modeled to be (cross-sectionally) predicted by each ANPS scale, which was significantly related to one of the (sub-)scales of the KSA-3 or the SDO scale in bivariate correlational analyses. ANPS variables were modeled as manifest variables based on the present sample size to ensure that the model converges. Also, age, gender (dummy-coded: 0 = men, 1 = women), and education (dummy-coded: 0 = no or some kind of school degree, 1 = university/university of applied sciences degree) were included in the model as manifest variables. Similarly, in the second SEM, the higher-order value types Openness to Change, Conservation, Self-Transcendence, and Self-Enhancement were modeled as higher-order factors with the sub-factors being the nine value types (Tradition was excluded). The four higher-order latent factors were modeled to be (cross-sectionally) predicted by each ANPS scale, which was significantly related to one of the respective value type scales of the PVQ in bivariate correlational analyses. As for the first SEM, ANPS variables were included as manifest variables in the model. Additionally, age, gender, and education were included as manifest variables in the model. For both SEMs, the Maximum Likelihood Estimator was used. In order to include gender as a dummy-variable in the models, the SEM analyses are based on individuals stating to identify as either a man or a woman (N = 623).

A final SEM, in which latent factors based on both ideological attitudes and personal value types are (cross-sectionally) predicted by the ANPS can be found in the S1 File. Of note: in the preregistration it is mentioned that a Principal Component Analysis (PCA) on the KSA-3, SDO, and value type scales would be conducted to extract component scores for each participant, which can, in turn, be (cross-sectionally) predicted by means of regression analyses. Instead of separate PCA and regression analyses, it was decided to compute Exploratory Factor Analysis and SEMs to model latent factors across KSA-3, SDO, and value type scales in the S1 File. Moreover, the separate analyses for RWA and SDO versus value types in two separate models were not preregistered but deemed important given the differential backgrounds of ideological attitudes versus personal value types, which are rooted in different theories.

## Results

### Descriptive statistics and associations with age, gender, and education

Descriptive statistics and differences between men and women are presented in Table 1. Women scored higher than men on FEAR, CARE, and SADNESS of the ANPS as well as on Conformity, Benevolence, and Universalism. Men scored higher than women on the KSA-3 total scale and its subscale Authoritarian Aggression, as well as SDO, and the Power scale.

**Table 1:** Descriptive statistics on variables of main interest and differences between men and women.

	Total Sample (N = 626)		Men (n = 403)		Women (n = 220)		Differences between men and women
**ANPS**							
SEEKING	2.89	0.35	2.89	0.37	2.89	0.32	t(518.1) = 0.32, p = 0.752
FEAR	2.67	0.52	2.62	0.49	2.76	0.55	t(412.9) = -3.21, p = 0.001
CARE	2.85	0.44	2.75	0.43	3.03	0.41	t(621) = -8.05, p < 0.001
ANGER	2.48	0.49	2.48	0.51	2.46	0.45	t(621) = 0.43, p = 0.664
PLAY	2.85	0.40	2.87	0.39	2.83	0.42	t(621) = 1.19, p = 0.234
SADNESS	2.45	0.43	2.39	0.43	2.56	0.39	t(494.8) = -5.13, p < 0.001
**KSA-3**							
Total	2.13	0.63	2.17	0.66	2.04	0.56	t(515.9) = 2.54, p = 0.011
Authoritarian Aggression	2.21	0.85	2.33	0.88	1.99	0.76	t(511.6) = 4.97, p < 0.001
Authoritarian Submission	2.30	0.86	2.30	0.90	2.31	0.78	t(507.2) = -0.16, p = 0.871
Conventionalism	1.87	0.71	1.89	0.72	1.83	0.70	t(621) = 1.02, p = 0.310
**SDO scale**							
SDO	2.13	0.86	2.24	0.93	1.95	0.69	W = 51952, p < 0.001
**PVQ**							
Self-Direction	4.57	0.89	4.57	0.94	4.56	0.80	t(515.1) = 0.25, p = 0.805
Stimulation	3.51	1.18	3.54	1.19	3.45	1.16	t(621) = 0.83, p = 0.404
Hedonism	4.13	1.01	4.19	1.00	4.03	1.02	t(621) = 1.92, p = 0.056
Achievement	4.01	1.10	4.05	1.14	3.95	1.02	t(494.2) = 1.15, p = 0.251
Power	3.32	1.05	3.46	1.09	3.06	0.91	t(519.7) = 4.84, p < 0.001
Security	3.92	1.09	3.88	1.10	3.99	1.05	t(621) = -1.28, p = 0.203
Conformity	3.53	1.11	3.46	1.11	3.66	1.09	t(621) = -2.13, p = 0.034
Benevolence	5.03	0.78	4.94	0.78	5.20	0.76	W = 35070, p < 0.001
Universalism	5.15	0.67	5.08	0.71	5.29	0.58	W = 36660, p < 0.001

ANPS = Affective Neuroscience Personality Scales, KSA-3 = Short Scale on Authoritarianism, SDO = Social Dominance Orientation, PVQ = Portraits Value Questionnaire; range ANPS: 1–4, range KSA-3: 1–5, range SDO: 1–7, range PVQ: 1–6; descriptive statistics for the group of individuals not identifying as a man or a woman are not presented due to the small subsample size.

In the total sample, age was significantly related to FEAR (rho = -0.24, p < 0.001), SADNESS (rho = -0,14, p < 0.001), the total KSA-3 scale (rho = -0.16, p < 0.001) as well as Authoritarian Aggression (rho = -0.17, p < 0.001) and Authoritarian Submission (rho = -0.19, p < 0.001), Stimulation (rho = -0.14, p < 0.001), Hedonism (rho = -0.16, p < 0.001), Achievement (rho = -0.17, p < 0.001), Power (rho = -0.18, p < 0.001), and Security (rho = -0.09, p = 0.020).

Differences between individuals with different educational backgrounds were found in SEEKING (W = 37709, p = 0.013), FEAR (t(624) = 4.70, p < 0.001), PLAY (t(624) = -2.16, p = 0.031), SADNESS (t(624) = 4.20, p < 0.001), the KSA-3 total scale (t(624) = 3.20, p = 0.001), the Authoritarian Aggression (t(624) = 3.22, p = 0.001) and Authoritarian Submission (t(624) = 2.73, p = 0.007) scales, and Security (t(624) = 2.03, p = 0.042).

### Correlational analysis

**Fig 2:**
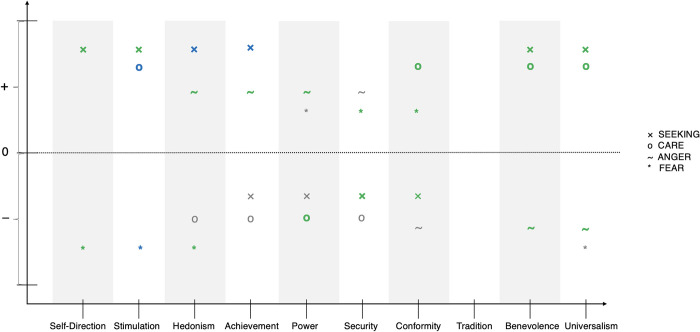
Significant correlations related to the initially reported hypotheses on value type relations. Green indicates that a hypothesis was supported by the data, grey indicates that a hypothesis was not supported by the data, blue indicates that relations were found that were not hypothesized (only related to SEEKING, CARE, ANGER, FEAR).

The KSA-3 scales and the SDO scale were positively correlated: KSA-3 total: rho = 0.47, p < 0.001, KSA-3 Authoritarian Aggression: rho = 0.40, p < 0.001, KSA-3 Authoritarian Submission: rho = 0.30, p < 0.001, KSA-3 Conventionalism: rho = 0.40, p < 0.001. Correlations of the ANPS with the KSA-3, SDO, and PVQ scales are presented in Table 2. Results in light of the proposed hypotheses related to value types are also graphically depicted in Fig 2.

**Table 2:** Zero-order correlations of the ANPS with the KSA-3, SDO, and PVQ scales.

	SEEKING	FEAR	CARE	ANGER	PLAY	SADNESS
KSA-3 Total	-0.09	0.08	-0.06	0.09	-0.05	0.06
	0.022	0.039	0.135	0.027	0.225	0.140
KSA-3 Authoritarian Aggression	-0.06	0.11	-0.13	0.15	-0.07	0.03
	0.144	0.007	< 0.001	< 0.001	0.066	0.385
KSA-3 Authoritarian Submission	-0.07	0.07	0.00	0.04	0.00	0.05
	0.097	0.096	0.948	0.344	0.913	0.216
KSA-3 Conven-tionalism	-0.09	0.01	0.00	0.01	-0.04	0.06
	0.018	0.791	0.974	0.745	0.367	0.160
*SDO*	*-0*.*06*	*-0*.*06*	*-0*.*21*	*0*.*09*	*-0*.*09*	*-0*.*07*
	*0*.*155*	*0*.*121*	*< 0*.*001*	*0*.*019*	*0*.*029*	*0*.*103*
Self-Direction	0.45	-0.16	0.05	0.00	0.13	-0.13
	< 0.001	< 0.001	0.261	0.920	0.002	0.001
Stimulation	0.46	-0.25	0.14	-0.04	0.40	-0.14
	< 0.001	< 0.001	< 0.001	0.382	< 0.001	< 0.001
Hedonism	0.17	-0.16	0.04	0.09	0.49	-0.11
	< 0.001	< 0.001	0.312	0.023	< 0.001	0.008
Achievement	0.25	0.07	0.00	0.20	0.08	0.03
	< 0.001	0.098	0.971	< 0.001	0.050	0.491
Power	0.07	-0.01	-0.19	0.26	-0.01	-0.03
	0.079	0.837	< 0.001	< 0.001	0.717	0.527
Security	-0.08	0.20	0.07	0.08	-0.03	0.10
	0.041	< 0.001	0.064	0.054	0.490	0.009
Conformity	-0.16	0.23	0.08	-0.01	-0.13	0.13
	< 0.001	< 0.001	0.036	0.715	< 0.001	0.001
Benevolence	0.19	0.00	0.55	-0.11	0.27	0.14
	< 0.001	0.976	< 0.001	0.005	< 0.001	< 0.001
*Universalism*	*0*.*27*	*-0*.*02*	*0*.*39*	*-0*.*10*	*0*.*14*	*0*.*00*
	*< 0*.*001*	*0*.*708*	*< 0*.*001*	*0*.*009*	*< 0*.*001*	*0*.*925*

KSA-3 = Short Scale on Authoritarianism, SDO = Social Dominance Orientation; italics represent Spearman correlations; only uncorrected p-values are presented; manually applying a Bonferroni-Holm correction for the 14×6 correlations leads to only the correlations with a p-value < 0.001 still being significant.

### Structural equation models

#### Ideological attitudes

**Fig 3:**
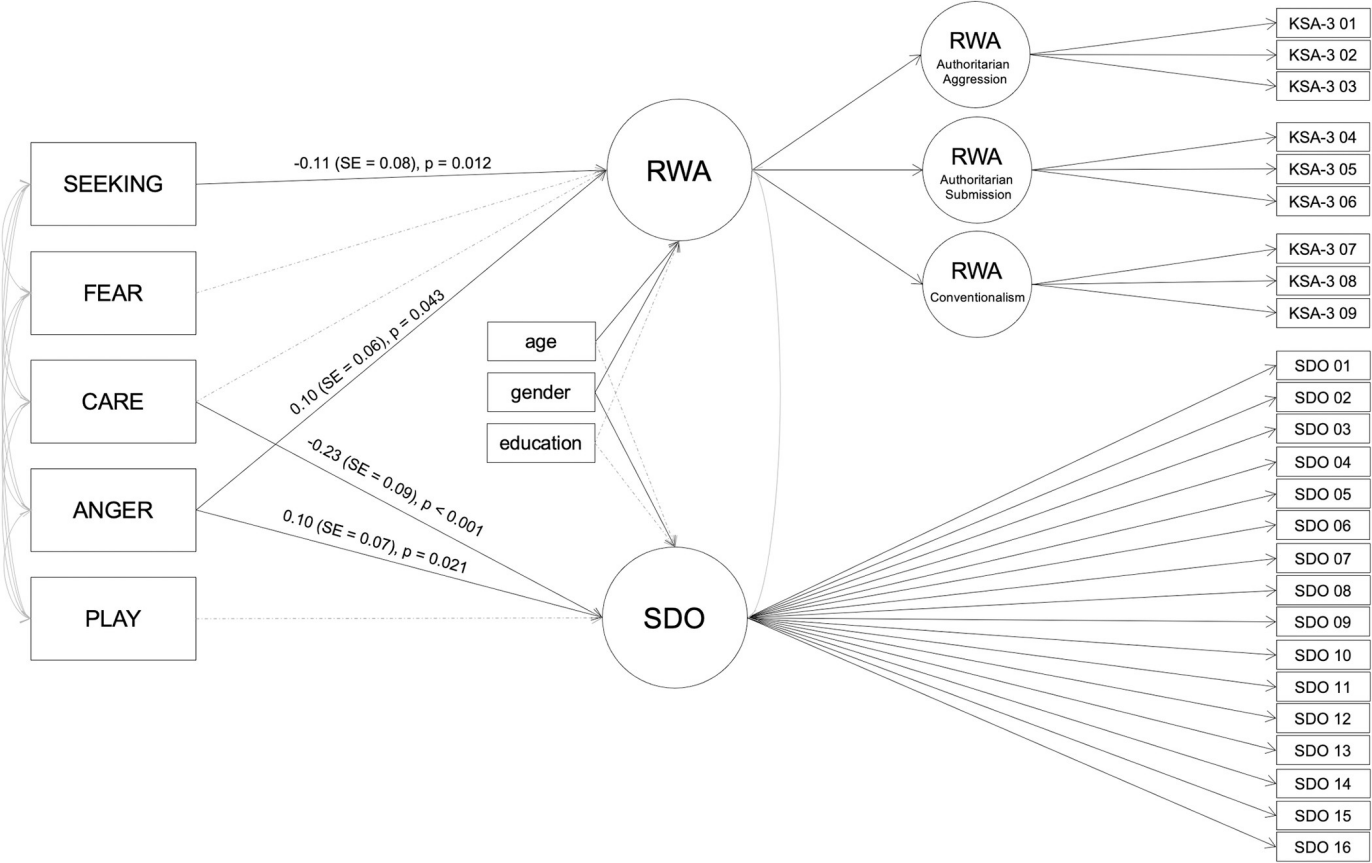
Results of the structural equation model on predicting RWA and SDO by the ANPS scale scores. Dashed lines indicate associations which were modeled but turned out to be non-significant; variances are not included in this figure for easier interpretability; similarly, standardized estimates are only presented for the significant ANPS-RWA/SDO relations for easier readability.

Fig 3 presents the significant associations between the ANPS and RWA as well as SDO based on the SEM. As can be seen from this figure, SEEKING was negatively related to RWA, while ANGER was positively associated with RWA. CARE was negatively related to SDO and ANGER was positively associated with SDO. More detailed results can be found in the S1 File.

#### Personal value types

**Fig 4:**
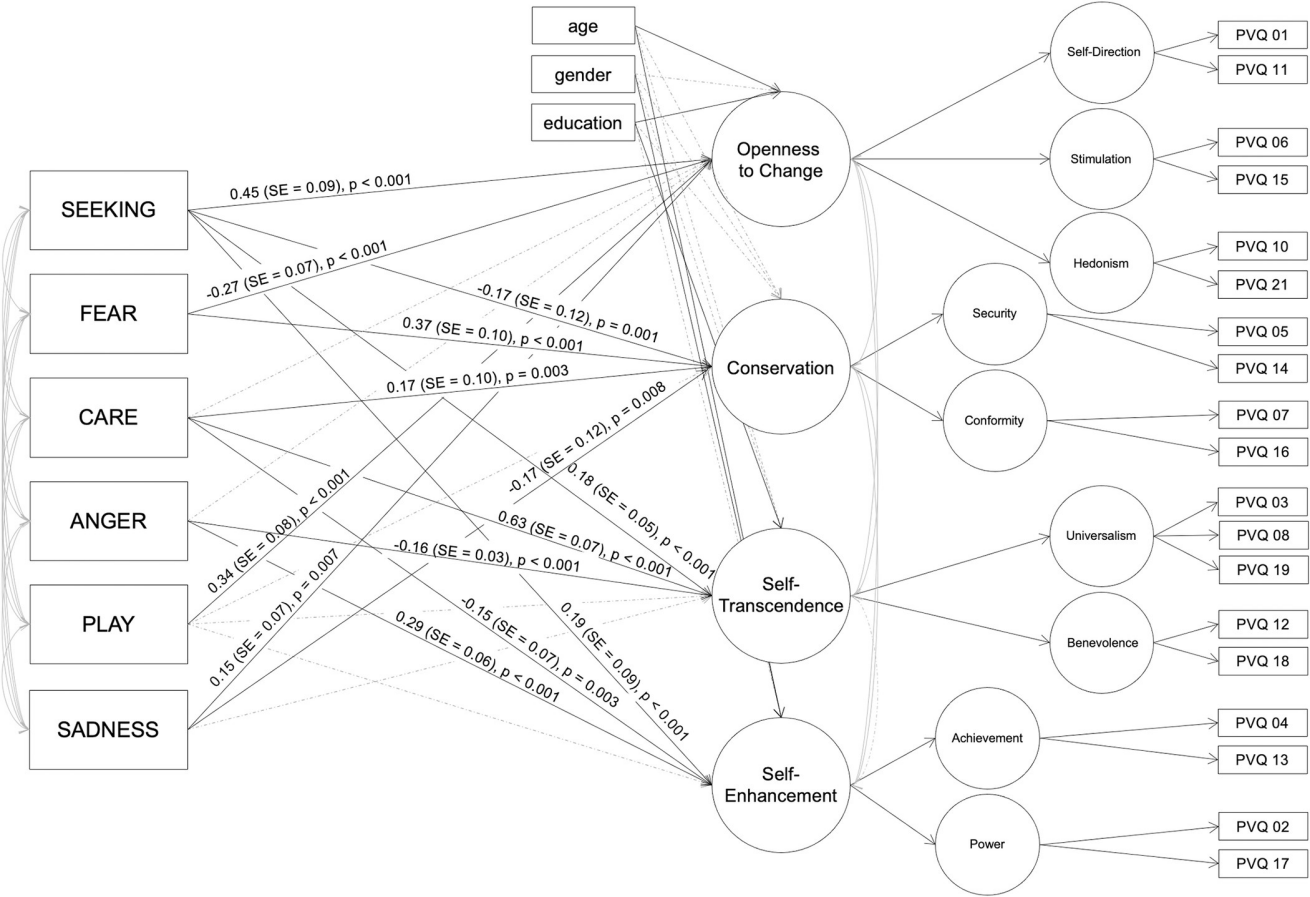
Results of the structural equation model on predicting higher-order value types by the ANPS scale scores. Dashed lines indicate associations which were modeled but turned out to be non-significant; variances are not included in this figure for easier interpretability; similarly, standardized estimates are only presented for the significant ANPS-value type relations for easier readability.

Fig 4 presents the significant associations between the ANPS and the four higher order value types based on the SEM. As can be seen from this figure, the higher order Openness to Change value type is positively related to SEEKING, PLAY, and SANDESS, and it is negatively related to FEAR. The higher order value type Conservation is positively related to FEAR and CARE and negatively to SEEKING and SADNESS. Furthermore, the Self-Transcendence value type is positively associated with SEEKING, and CARE, as well as negatively with ANGER. Finally, the Self-Enhancement value type is positively related to SEEKING, and ANGER, and negatively to CARE. More detailed results on this SEM can be found in the S1 File.

## Discussion

The present work was conducted in order to unravel the personality trait associations of ideological attitudes and personal value types against the background of the ANT. Based on theory and research, the focus of the present research was on relations of SEEKING, FEAR, CARE, and ANGER with ideological attitudes and personal value types. The relations with other PETs were investigated exploratory. The discussion will mostly focus on the relations found with the previously mentioned four PETs, accordingly. First, the results will be revisited and discussed in light of hypotheses of the present work, previous research on trait theories, RWA, SDO, and values, as well as in light of research on emotion theories beyond ANT. Afterward, implications of the present findings for future studies are provided. For this, a specific focus is put on implications for research in the field of political neuroscience. Finally, the limitations of the present work are described before a conclusion is drawn.

### Discussion in light of hypotheses and previous research and theories

To begin with and as expected in the first hypothesis, RWA and SDO were positively related with moderate to large effect sizes; based on transferring the effect size categorizations by Cohen [57,58] from Pearson to Spearman correlations. More specifically, the shared variance between the two ideological attitudes was 0.47×0.47, or 22.09%. This result is in line with theory [7,8], previous research in other German-speaking samples [25,37,59,60], and our first hypothesis indicating that RWA and SDO are positively related but separable constructs. This assumption is also underlined by differences in the relations of both ideological attitudes with PETs.

Regarding associations between ideological attitudes and PETs, the results of the SEM will be discussed in the following. This is because the SEM enables the investigation of relations between PETs and both ideological attitudes at the same time. SEM results revealed that while lower SEEKING and higher ANGER were related to higher RWA, lower CARE and higher ANGER were associated with higher SDO. The association of SEEKING with RWA is in line with hypothesis 2 of the present work. However, the additional positive relation of SEEKING with SDO proposed in hypothesis 2 was not observed in the SEM. Moreover, hypotheses 3 and 4 on relations of ANGER and CARE with SDO were supported by the present data. Finally, hypothesis 5 was not supported by the data, since at least in the SEM no significant relation between FEAR and RWA was observed.

The relation of SEEKING with RWA fits to results found in previous literature: SEEKING is thought to be most strongly related to the Big Five trait Openness [21], which is in turn negatively related to RWA as proposed in theoretical models and as underlined by results of empirical studies [31,32]. High SEEKING in terms of the ANT describes individuals who tend to show explorative and “appetitive” behaviors [16]. Individuals scoring high in SEEKING might embrace the new experiences, also related to societal orders and functioning. Thus, they might not enjoy following old traditions and conventional ways of living. This explanation fits with the finding that SEEKING was especially negatively correlated with the RWA facet conventionalism in the present work. Further, the negative relation between SEEKING and RWA fits a study on emotions and RWA. The results of this previous work reveal negative relations between experiencing positive emotions and RWA [61]. Positive emotions were assessed by the Positive and Negative Affect Schedule (PANAS) and included among others being enthusiastic, interested, and active, which fit the description of SEEKING (but note that SEEKING is derived from a trait approach and that positive emotionality as assessed via the PANAS also comprised other emotions). Other work, however, reports non-significant relations between RWA and affect [62].

Next, ANGER being positively related to both RWA and SDO also aligns with findings of previous research: Previous research in emotions showed that RWA was positively related to experiencing negative emotions, including among others feeling irritable and hostile, which aligns with the description of high ANGER scores [61]. Of note, in the present work, ANGER was specifically related to the Authoritarian Aggression scale of RWA. Aside from the positive relation of ANGER with SDO, CARE was negatively related to SDO. These two associations with SDO also fit perfectly to the Dual-Process Motivational Model constituting low Agreeableness to be linked to high SDO. Both low CARE and high ANGER are related to low Agreeableness [21]. It also seems logically correct, that individuals who like to take care of others (high CARE) do not prefer hierarchies within society. Individuals who are hotheaded and get angry easily and who tend to strive to protect their own resources (high ANGER items in the ANPS) tend to prefer hierarchies and to keep certain groups in their place (high SDO) [11,16,26,48].

**Fig 5:**
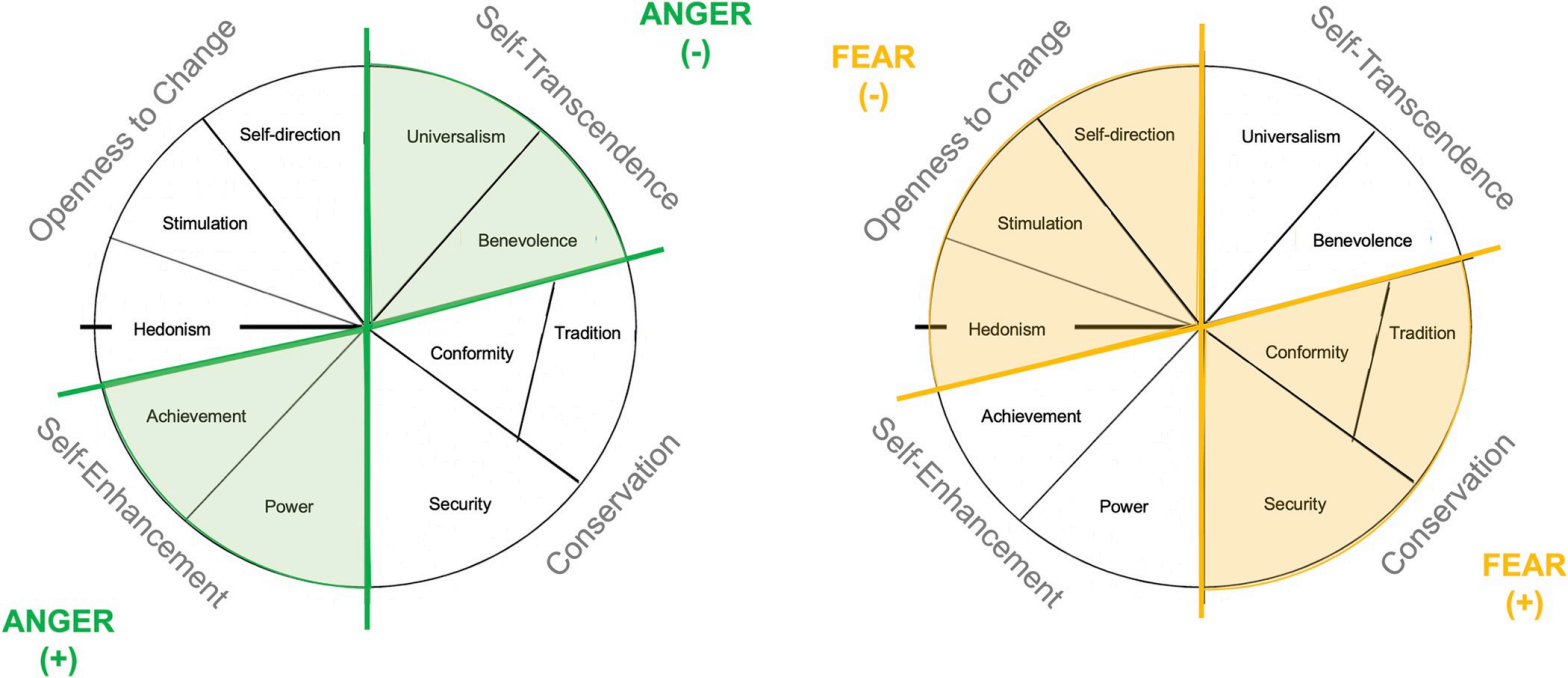
Main results on relations of ANGER and FEAR with the individual value types according to Schwartz.

In relation to associations between PETs and personal value types, specifically results related to FEAR and ANGER are discussed. This is because these revealed–from our perspective–the most consistent results on relations with value type scores across correlational and SEM analyses; see Fig 5.

FEAR was associated with the higher-order value type dimension ranging from Conservation to Openness to Change. More specifically, while Conservation was positively related to FEAR, Openness to Change exhibited a negative relation with FEAR. Individuals describing themselves as being anxious, nervous, easily frightened, and worrying a lot (high FEAR) [11,48,49] seem to prefer security and safety as well as stability in society (high scores in Security value type) and seem to like following social norms and orders (high scores in Conformity value type). Putatively, stability and having a blueprint of rules to follow give individuals high in FEAR a feeling of control and safety. This new hypothesis should be tested in forthcoming studies. These findings also fit some results related to experiencing emotions: The higher-order value type Openness to Change has been positively related to positive affect, i.e. experiencing more positive versus negative emotions in previous work [63]. Moreover, another study among others reports Stimulation and Self-Direction (belonging to the Openness to Change higher-order value type) to be negatively related to anxiousness, and Stimulation to be negatively related to depressivity (assessed by the Personality Inventory for the DSM-5). The same study also reports positive relations of Security (part of the higher-order-value type Conservation) with anxiousness also fitting to the present findings [64]. Once another study found among others that hedonism (part of the Openness to Change higher-order value type) is negatively related to depression and anxiety, and that Stimulation is negatively related to anxiety [65].

ANGER was related to the value type dimension spanning from Self-Transcendence to Self-Enhancement. In detail, ANGER was positively related to Self-Enhancement and negatively to Self-Transcendence. Since ANGER of the ANPS is not only related to being easily irritated and angry but also to protecting one’s own resources according to ANT [16], it seems reasonable that individuals scoring low in ANGER follow value types related to Universalism and Benevolence, thus, the well-being of others instead of themselves. This finding again fits results on experiencing certain emotions: the higher-order Self-Enhancement value type and especially the power value type have been related to experiencing anger [66], contempt, hostility, hatred, and pride [67]. Moreover, both Power and achievement were found to positively relate to hostility (assessed by the Personality Inventory for the DSM-5) [64].

### Implications based on the Affective Neuroscience Theory

Next to discussing the present findings in relation to previous literature, including the literature on relations of RWA, SDO, and value types with personality traits and experiencing certain emotions, it is also of considerable interest to discuss implications of the present work for future studies. For this, the biological correlates of the ANT and PETs, accordingly, are discussed more in-depth. Thereby, hypotheses on biological correlates of political views, like RWA, SDO, and value types, can be derived for forthcoming studies.

Neuroanatomical, neurochemical, pharmacological, and physiological brain research on mammals was conducted by Jaak Panksepp and summarized in the ANT providing documentation of subcortical emotional systems in the mammalian brain as the basis of emotional behavior [15,16]. According to the ANT, these systems have been conserved across the mammalian brain and can also be found in the human brain. As such, they are thought to be related to the PETs investigated in the present work [11,48]. In more, detail, and although the primary emotional systems can be found in all human brains according to ANT, individual differences in their neuroanatomy, size, and functionality could be related to individual differences in personality [68]. In line with this, PETs have been linked to biological variables, such as brain structure [69], resting state functional connectivity of certain brain areas [70], and genetic markers [69,71–73] in humans. Given the well-explored biological correlates of PETs–at least in mammals–links of individual differences in ideological attitudes and personal value type scores to individual differences in PETs might reveal putative links to certain brain areas and neurotransmitters/-peptides, accordingly.

For instance, the brain neuroanatomy and neurotransmitters/-peptides related to SEEKING according to the ANT are presented in Table 3. Future research can use the brain areas and neurotransmitters/-peptides linked to SEEKING in order to investigate putative biological predispositions related to RWA, and more specifically, its conventionalism facet. The brain neuroanatomy and neurotransmitters/-peptides related to CARE and ANGER are also presented in Table 3 and might provide information for hypotheses building in forthcoming research projects striving to unravel which specific biological markers are related to SDO (CARE, ANGER) as well as RWA (ANGER). Importantly, ANGER is among others related to the medial amygdala according to the ANT, and CARE is related to the anterior cingulate (see Table 3). This makes these two brain areas interesting candidates to be studied in relation to individual differences in SDO (anterior cingulate, medial amygdala) and RWA (medial amygdala) in forthcoming studies in the field of political neuroscience. Indeed, a study using structural magnetic resonance imaging found negative relations between the volume of the anterior cingulate and conservatism as well as positive relations between (right) amygdala volume and conservatism [74]. Conservatism is known to be positively related to both RWA and SDO [75], further underlining the importance of those brain areas for both ideological attitudes. Moreover, Weissflog et al. [76] reported differences in the activity of the anterior cingulate cortex (more specifically, the error-related negativity) in a Go/NoGo task being associated with both attitudes towards social change (RWA) and attitudes towards social (in)equalities (putatively being related to SDO). These findings further support the importance of this brain area, among others in SDO. Finally, since both RWA and (to a lesser extent) SDO are in parts heritable [77–80] the findings of the present study might additionally support forthcoming studies in investigating which specific genetic markers might be related to those constructs. Thus, based on the present findings, researchers can follow the candidate gene approach and formulate hypotheses to investigate specific genetic polymorphisms and their relations to individual differences in RWA and SDO in a hypotheses-driven way; instead of in hypotheses-free Genome-Wide Association Studies (GWAS). Accordingly, the present findings might set a new starting point to unravel the complex (molecular genetic) relations of brain anatomy and function with RWA, SDO, and political attitudes, as well as ideologies more broadly in future research projects. This new starting point seems important because previous literature on relations between genetics and ideology is inconclusive. This is illustrated, for example, in the GWAS by Hatemi et al. [81]. In this realm, also the review work by Dawes and Weinschenk [82] on the genetic relations of political ideology, being closely linked to ideological attitudes, is of interest. It gives an in-depth explanation of promises and perils of genetic association studies in the field of political psychology.

**Table 3:** Primary emotional traits/systems and their brain neuroanatomy and neurotransmitters/neuropeptides.

Primary Emotional Trait (System)	Brain Neuroanatomy Related to the System	Some Neurotransmitters/ Neuropeptides Related to the System
SEEKING	Nucleus Accumbens to ventral tegmental area, mesolimbic and mesocortical outputs, lateral hypothalamus to periaqueductal gray	Dopamine (+), glutamate (+), opioids (+), neurotensin (+), and orexin (+)
FEAR	Central and lateral amygdala to medial hypothalamus and dorsal periaqueductal gray	Glutamate (+), corticotropin releasing factor/hormone (+), cholecystokinin (+), alpha-melanocyte stimulating hormone (+), oxytocin (-)
CARE	Anterior cingulate, bed nucleus of stria terminalis, preoptic area, ventral tegmental area, periaqueductal gray	Oxytocin (+), prolactin (+), dopamine (+), opioids (+/−)
ANGER (RAGE)	Medial amygdala to bed nucleus of stria terminalis; medial and perifornical hypothalamus to periaqueductal gray	Substance P (+), acetylcholin (+), glutamate (+)

+ excitatory effects; − inhibitory effects; table content based among others on Montag and Davis [68], which is based on Panksepp [16].

As for RWA and SDO, also personal value types are in parts heritable [83]. Thus, not only can the present findings support investigations in the field of political neuroscience and ideological attitudes. Besides, the present findings might also serve as a roadmap to build hypotheses to study biological correlates of personal value types; putatively with a focus on–for example–the glutamate (FEAR, ANGER; see Table 3) or oxytocin system (FEAR; see Table 3) or the acetylcholine system (ANGER; see Table 3). Related to brain function and structure, also other empirical studies are of interest, providing a somewhat different picture: one study found that processing related to the higher-order value type Self-Transcendence was associated with higher brain activity in the dorsomedial and ventromedial prefrontal cortices compared to processing related to the higher-order Self-Enhancement value type [84]. Another study reports positive relations between Hedonism (part of the higher-order value type Openness to Change or Self-enhancement) and volume of the globus pallidus [85]. Since psychological constructs, such as value types, are complex constructs, several brain areas and systems will be related to individual differences in value types. An investigation of a combination of brain areas, such as those derived from ANT and those based on previous empirical research, and their co-functioning in relation to value types might be an interesting new research avenue.

### Limitations of the present work

Finally, some limitations and shortcomings of the present study must be acknowledged. First, putative relations of the variables investigated in this work with biological variables can only be assumed based on theory and previous empirical research. Such associations can, however, not be tested using the present data. Furthermore, because of the cross-sectional study design, causal relations cannot be tested with the present data. In addition, the fit values of the SEMs, which were overall non-satisfactory according to acknowledged cut-off scores [86], need to be mentioned. Conducting changes in the models according to modification indices, such as allowing correlations between certain items, did not help to overcome the problem. Only including RWA, SDO, and the four higher-order value types, respectively, as manifest variables in the models did improve the fit values meaningfully. Doing so did barely change the main findings of the present study: Relations of SEEKING and ANGER with RWA, of ANGER and CARE with SDO, of FEAR with the higher-order value type dimension Conservation to Openness to Change, and of ANGER with the higher-order value type dimension Self-Transcendence to Self-Enhancement remained significant. It was decided to present SEMs based on latent ideological attitudes variables and value type dimensions, nevertheless. This decision was based on the fact that all latent variables were modeled based on previous literature (see, for example, Beierlein et al. [25], who report a satisfactory fit of the higher-order RWA model using the same questionnaire as applied in the present work in a German-speaking sample). Additionally, the fact that main results did not change based on whether latent or manifest variables were used, did support this decision. Another limitation is that the present study investigates relations only in one German sample. Thus, future studies testing the replicability and generalizability of the present results not only in other German samples but also in samples with other sociocultural backgrounds are necessary. At last, the overall small effect sizes found in the present study for relations of PETs with ideological attitudes and personal value types need to be discussed. These small effect sizes point toward other dispositional as well as environmental (social, situational, etc.) factors additionally and interactively being linked to the complex phenomena of ideological attitudes and scores in personal value types. Similarly, also biological correlates of both ideological attitudes and value types will most likely be highly complex and comprise more than one brain area or one neurotransmitter/-peptide.

## Conclusions

The present study results indicate that especially the personality traits SEEKING (negatively) and ANGER (positively) are associated with RWA. CARE (negatively) and ANGER (positively) seem to be most strongly related to SDO. FEAR and ANGER seem to be most strongly linked to the higher-order value type dimensions spanning from Conservation to Openness to Change and from Self-Transcendence to Self-Enhancement, respectively. Based on these results, forthcoming studies may investigate putative biological correlates of ideological attitudes and the two higher-order value type dimensions focusing on brain areas and neurotransmitters/-peptides as well as genetic polymorphisms related to these PETs.

## Supporting information

S1 File This file contains information on data cleaning as well as additional results. (PDF)
