# Supplementary material for: Linking primary emotional traits to ideological attitudes and personal value types
Source: PLoS One. 2023 Jan 3;18(1):e0279885. doi: 10.1371/journal.pone.0279885 (PMC9810181; doi:10.1371/journal.pone.0279885)
Supplement: S1 File — (PDF) [file pone.0279885.s001.pdf]

**Supplementary Material**

**Linking Primary Emotional Traits to Ideological Attitudes and Personal Value Types**

Cornelia Sindermann, Christopher Kannen, Christian Montag

## Data Cleaning

In total, N = 808 individuals completed the present online survey. Of these, n = 134 were excluded because of missing data in variables of interest for the present work. The high number of individuals with missing data is due to the fact that completing the Affective Neuroscience Personality Scales (ANPS) was not mandatory. One (n = 1) additional participant was excluded from final analyses because he/she was younger than 18 years. No participant needed to be excluded because of not being eligible to vote in the general German elections in 2021. However, n = 3 additional individuals were excluded because of being suspected to respond carelessly; they chose the same response option throughout at least one of the pages on which items of the Social Dominance Orientation (SDO) scale, the Portraits Value Questionnaire (PVQ) or the ANPS were presented. Note that responding to the KSA-3 items assessing Right-Wing Authoritarianism (RWA) with the same response option was not deemed to indicate careless responding. Finally, n = 44 individuals were excluded from final analyses because of failing the attention check item, in which participants were explicitly asked to choose a certain response option. This led to the final sample of N = 626 participants.

## Correlations Between Variables of Main Interest

## Supplementary Table 1. Zero-Order correlations between variables of main interest.

| | 1 | 2 | 3 | 4 | 5 | 6 | 7 | 8 | 9 | 10 | 11 | 12 | 13 | 14 | 15 | 16 | 17 | 18 | 19 |
|---|---|---|---|---|---|---|---|---|---|---|---|---|---|---|---|---|---|---|---|
| 1. SEEKING | | | | | | | | | | | | | | | | | | | |
| 2. FEAR | -0.24 | | | | | | | | | | | | | | | | | | |
| | <0.001 | | | | | | | | | | | | | | | | | | |
| 3. CARE | 0.22 | 0.06 | | | | | | | | | | | | | | | | | |
| | <0.001 | 0.108 | | | | | | | | | | | | | | | | | |
| 4. ANGER | -0.01 | 0.30 | -0.06 | | | | | | | | | | | | | | | | |
| | 0.823 | <0.001 | 0.131 | | | | | | | | | | | | | | | | |
| 5. PLAY | 0.38 | -0.30 | 0.33 | -0.08 | | | | | | | | | | | | | | | |
| | <0.001 | <0.001 | <0.001 | 0.057 | | | | | | | | | | | | | | | |
| 6. SADNESS | -0.27 | 0.68 | 0.21 | 0.26 | -0.20 | | | | | | | | | | | | | | |
| | <0.001 | <0.001 | <0.001 | <0.001 | <0.001 | | | | | | | | | | | | | | |
| 7. KSA-3 Total | -0.09 | 0.08 | -0.06 | 0.09 | -0.05 | 0.06 | | | | | | | | | | | | | |
| | 0.022 | 0.039 | 0.135 | 0.027 | 0.225 | 0.140 | | | | | | | | | | | | | |
| 8. KSA-3 AA | -0.06 | 0.11 | -0.13 | 0.15 | -0.07 | 0.03 | 0.82 | | | | | | | | | | | | |
| | 0.144 | 0.007 | <0.001 | <0.001 | 0.066 | 0.385 | <0.001 | | | | | | | | | | | | |
| 9. KSA-3 AS | -0.07 | 0.07 | 0.00 | 0.04 | 0.00 | 0.05 | 0.81 | 0.50 | | | | | | | | | | | |
| | 0.097 | 0.096 | 0.948 | 0.344 | 0.913 | 0.216 | <0.001 | <0.001 | | | | | | | | | | | |
| 10. KSA-3 Con. | -0.09 | 0.01 | 0.00 | 0.01 | -0.04 | 0.06 | 0.71 | 0.39 | 0.35 | | | | | | | | | | |
| | 0.018 | 0.791 | 0.974 | 0.745 | 0.367 | 0.160 | <0.001 | <0.001 | <0.001 | | | | | | | | | | |
| 11. SDO | *-0.06* | *-0.06* | *-0.21* | *0.09* | *-0.09* | *-0.07* | *0.47* | *0.40* | *0.30* | *0.40* | | | | | | | | | |
| | *0.155* | *0.121* | *<0.001* | *0.019* | *0.029* | *0.103* | *<0.001* | *<0.001* | *<0.001* | *<0.001* | | | | | | | | | |
| 12. Self-Direction | 0.45 | -0.16 | 0.05 | 0.00 | 0.13 | -0.13 | -0.16 | -0.09 | -0.10 | -0.19 | *-0.05* | | | | | | | | |
| | <0.001 | <0.001 | 0.261 | 0.920 | 0.002 | 0.001 | <0.001 | 0.020 | 0.013 | <0.001 | *0.198* | | | | | | | | |

| | | | | | | | | | | | | | | | | | | | |
|---|---|---|---|---|---|---|---|---|---|---|---|---|---|---|---|---|---|---|---|
| 13. Stimulation | 0.46 | -0.25 | 0.14 | -0.04 | 0.40 | -0.14 | -0.01 | -0.03 | 0.03 | -0.02 | *0.05* | 0.41 | | | | | | | |
| | <0.001 | <0.001 | <0.001 | 0.382 | <0.001 | <0.001 | 0.857 | 0.419 | 0.398 | 0.600 | *0.244* | <0.001 | | | | | | | |
| 14. Hedonism | 0.17 | -0.16 | 0.04 | 0.09 | 0.49 | -0.11 | 0.02 | 0.03 | 0.06 | -0.07 | *0.02* | 0.16 | 0.45 | | | | | | |
| | <0.001 | <0.001 | 0.312 | 0.023 | <0.001 | 0.008 | 0.689 | 0.382 | 0.137 | 0.077 | *0.648* | <0.001 | <0.001 | | | | | | |
| 15. Achievement | 0.25 | 0.07 | 0.00 | 0.20 | 0.08 | 0.03 | 0.18 | 0.17 | 0.17 | 0.07 | *0.17* | 0.09 | 0.11 | 0.10 | | | | | |
| | <0.001 | 0.098 | 0.971 | <0.001 | 0.050 | 0.491 | <0.001 | <0.001 | <0.001 | 0.097 | *<0.001* | 0.032 | 0.004 | 0.013 | | | | | |
| 16. Power | 0.07 | -0.01 | -0.19 | 0.26 | -0.01 | -0.03 | 0.32 | 0.36 | 0.23 | 0.14 | *0.30* | 0.06 | 0.10 | 0.14 | 0.55 | | | | |
| | 0.079 | 0.837 | <0.001 | <0.001 | 0.717 | 0.527 | <0.001 | <0.001 | <0.001 | <0.001 | *<0.001* | 0.136 | 0.010 | <0.001 | <0.001 | | | | |
| 17. Security | -0.08 | 0.20 | 0.07 | 0.08 | -0.03 | 0.10 | 0.50 | 0.43 | 0.39 | 0.35 | *0.14* | -0.13 | -0.23 | -0.03 | 0.14 | 0.20 | | | |
| | 0.041 | <0.001 | 0.064 | 0.054 | 0.490 | 0.009 | <0.001 | <0.001 | <0.001 | <0.001 | *<0.001* | 0.001 | <0.001 | 0.497 | <0.001 | <0.001 | | | |
| 18. Conformity | -0.16 | 0.23 | 0.08 | -0.01 | -0.13 | 0.13 | 0.41 | 0.33 | 0.33 | 0.29 | *0.03* | -0.28 | -0.33 | -0.21 | 0.14 | 0.13 | 0.48 | | |
| | <0.001 | <0.001 | 0.036 | 0.715 | <0.001 | 0.001 | <0.001 | <0.001 | <0.001 | <0.001 | *0.452* | <0.001 | <0.001 | <0.001 | <0.001 | 0.001 | <0.001 | | |
| 19. Benevolence | 0.19 | 0.00 | 0.55 | -0.11 | 0.27 | 0.14 | 0.01 | -0.05 | 0.04 | 0.04 | *-0.22* | 0.10 | 0.16 | 0.11 | 0.03 | -0.09 | 0.17 | 0.15 | |
| | <0.001 | 0.976 | <0.001 | 0.005 | <0.001 | <0.001 | 0.825 | 0.226 | 0.364 | 0.342 | *<0.001* | 0.011 | <0.001 | 0.008 | 0.389 | 0.019 | <0.001 | <0.001 | |
| *20. Universalism* | *0.27* | *-0.02* | *0.39* | *-0.10* | *0.14* | *0.00* | *-0.24* | *-0.25* | *-0.12* | *-0.19* | *-0.48* | *0.25* | *0.23* | *0.08* | *-0.06* | *-0.21* | *-0.06* | *-0.08* | *0.42* |
| | *<0.001* | *0.708* | *<0.001* | *0.009* | *<0.001* | *0.925* | *<0.001* | *<0.001* | *0.002* | *<0.001* | *<0.001* | *<0.001* | *<0.001* | *0.059* | *0.111* | *<0.001* | *0.120* | *0.045* | *<0.001* |

KSA-3 = Short Scale on Authoritarianism, AA = Authoritarian Aggression, AS = Authoritarian Submission, Con. = Conventionalism, SDO = Social Dominance Orientation; Italics represent spearman correlations; only uncorrected p-values are presented; N = 626.

## Detailed Results on the Structural Equation Model on RWA and SDO

**Supplementary Table 2. Detailed Results of the Structural Equation Model.**

**Latent variables:**

| | | Estimate | SE | z | p | Std.lv | Std.all |
|---|---|---|---|---|---|---|---|
| Authoritarian Aggression | | | | | | | |
| | rwa_01 | 1.000 | | | | 0.697 | 0.732 |
| | rwa_02 | 1.125 | 0.077 | 14.597 | <0.001 | 0.784 | 0.698 |
| | rwa_03 | 1.054 | 0.073 | 14.425 | <0.001 | 0.734 | 0.687 |
| Authoritarian Submission | | | | | | | |
| | rwa_04 | 1.000 | | | | 0.785 | 0.681 |
| | rwa_05 | 0.940 | 0.069 | 13.654 | <0.001 | 0.738 | 0.749 |
| | rwa_06 | 0.884 | 0.068 | 12.994 | <0.001 | 0.694 | 0.670 |
| Conventionalism | | | | | | | |
| | rwa_07 | 1.000 | | | | 0.603 | 0.532 |
| | rwa_08 | 1.047 | 0.096 | 10.888 | <0.001 | 0.631 | 0.770 |
| | rwa_09 | 0.859 | 0.080 | 10.723 | <0.001 | 0.518 | 0.691 |
| SDO | | | | | | | |
| | sdo_01 | 1.000 | | | | 0.869 | 0.572 |
| | sdo_02 | 0.433 | 0.054 | 7.976 | <0.001 | 0.377 | 0.356 |
| | sdo_03 | 0.996 | 0.074 | 13.478 | <0.001 | 0.866 | 0.697 |
| | sdo_04 | 0.632 | 0.051 | 12.289 | <0.001 | 0.550 | 0.609 |
| | sdo_05 | 0.695 | 0.077 | 8.995 | <0.001 | 0.604 | 0.409 |
| | sdo_06 | 1.124 | 0.082 | 13.781 | <0.001 | 0.977 | 0.722 |
| | sdo_07 | 0.768 | 0.057 | 13.381 | <0.001 | 0.668 | 0.689 |
| | sdo_08 | 0.865 | 0.097 | 8.950 | <0.001 | 0.752 | 0.406 |
| | sdo_09 | 1.018 | 0.091 | 11.132 | <0.001 | 0.885 | 0.532 |
| | sdo_10 | 1.441 | 0.106 | 13.605 | <0.001 | 1.253 | 0.707 |
| | sdo_11 | 0.704 | 0.056 | 12.559 | <0.001 | 0.612 | 0.628 |
| | sdo_12 | 1.063 | 0.074 | 14.291 | <0.001 | 0.924 | 0.765 |
| | sdo_13 | 0.966 | 0.074 | 12.987 | <0.001 | 0.840 | 0.659 |
| | sdo_14 | 1.083 | 0.079 | 13.675 | <0.001 | 0.942 | 0.713 |
| | sdo_15 | 1.285 | 0.106 | 12.143 | <0.001 | 1.117 | 0.599 |
| | sdo_16 | 0.918 | 0.077 | 11.860 | <0.001 | 0.799 | 0.579 |
| RWA | | | | | | | |
| | Authoritarian Aggression | 1.000 | | | | 0.887 | 0.887 |
| | Authoritarian Submission | 0.889 | 0.091 | 9.775 | <0.001 | 0.699 | 0.699 |
| | Conventionalism | 0.654 | 0.078 | 8.429 | <0.001 | 0.671 | 0.671 |

**Regressions:**

| | | Estimate | SE | z | p | Std.lv | Std.all |
|---|---|---|---|---|---|---|---|
| RWA | | | | | | | |
| | SEEKING | -0.191 | 0.076 | -2.503 | 0.012 | -0.309 | -0.110 |
| | FEAR | 0.084 | 0.056 | 1.508 | 0.132 | 0.137 | 0.071 |
| | CARE | -0.056 | 0.069 | -0.805 | 0.421 | -0.090 | -0.040 |
| | ANGER | 0.123 | 0.061 | 2.026 | 0.043 | 0.199 | 0.097 |
| | Age | -0.007 | 0.003 | -2.300 | 0.021 | -0.011 | -0.121 |
| | Gender (0 = male) | -0.156 | 0.064 | -2.452 | 0.014 | -0.253 | -0.121 |
| | Education (0 = no/school degree) | -0.101 | 0.067 | -1.508 | 0.131 | -0.164 | -0.077 |
| SDO | | | | | | | |
| | CARE | -0.442 | 0.092 | -4.788 | <0.001 | -0.508 | -0.225 |

| | | | | | | |
|---|---|---|---|---|---|---|
| ANGER | 0.168 | 0.073 | 2.316 | 0.021 | 0.193 | 0.095 |
| PLAY | -0.005 | 0.085 | -0.054 | 0.957 | -0.005 | -0.002 |
| Age | -0.001 | 0.004 | -0.199 | 0.843 | -0.001 | -0.009 |
| Gender (0 = male) | -0.173 | 0.079 | -2.196 | 0.028 | -0.199 | -0.095 |
| Education (0 = no/school degree) | 0.033 | 0.083 | 0.401 | 0.689 | 0.038 | 0.018 |

**Covariances:**

| | | Estimate | SE | z | p | Std.lv | Std.all |
|---|---|---|---|---|---|---|---|
| SDO | | | | | | | |
| | RWA | 0.301 | 0.036 | 8.448 | <0.001 | 0.616 | 0.616 |

**Variances:**

| | Estimate | SE | z | p | Std.lv | Std.all |
|---|---|---|---|---|---|---|
| rwa_01 | 0.421 | 0.034 | 12.319 | <0.001 | 0.421 | 0.465 |
| rwa_02 | 0.645 | 0.049 | 13.260 | <0.001 | 0.645 | 0.512 |
| rwa_03 | 0.602 | 0.044 | 13.534 | <0.001 | 0.602 | 0.528 |
| rwa_04 | 0.713 | 0.055 | 13.015 | <0.001 | 0.713 | 0.536 |
| rwa_05 | 0.425 | 0.039 | 10.815 | <0.001 | 0.425 | 0.438 |
| rwa_06 | 0.594 | 0.045 | 13.316 | <0.001 | 0.594 | 0.552 |
| rwa_07 | 0.923 | 0.060 | 15.411 | <0.001 | 0.923 | 0.717 |
| rwa_08 | 0.273 | 0.030 | 9.120 | <0.001 | 0.273 | 0.407 |
| rwa_09 | 0.294 | 0.024 | 12.037 | <0.001 | 0.294 | 0.523 |
| sdo_01 | 1.558 | 0.092 | 16.895 | <0.001 | 1.558 | 0.673 |
| sdo_02 | 0.980 | 0.056 | 17.424 | <0.001 | 0.980 | 0.874 |
| sdo_03 | 0.793 | 0.049 | 16.176 | <0.001 | 0.793 | 0.514 |
| sdo_04 | 0.513 | 0.031 | 16.733 | <0.001 | 0.513 | 0.629 |
| sdo_05 | 1.820 | 0.105 | 17.338 | <0.001 | 1.820 | 0.833 |
| sdo_06 | 0.879 | 0.055 | 15.956 | <0.001 | 0.879 | 0.479 |
| sdo_07 | 0.492 | 0.030 | 16.238 | <0.001 | 0.492 | 0.525 |
| sdo_08 | 2.861 | 0.165 | 17.342 | <0.001 | 2.861 | 0.835 |
| sdo_09 | 1.980 | 0.116 | 17.034 | <0.001 | 1.980 | 0.717 |
| sdo_10 | 1.569 | 0.098 | 16.090 | <0.001 | 1.569 | 0.500 |
| sdo_11 | 0.575 | 0.035 | 16.637 | <0.001 | 0.575 | 0.606 |
| sdo_12 | 0.604 | 0.039 | 15.446 | <0.001 | 0.604 | 0.415 |
| sdo_13 | 0.918 | 0.056 | 16.453 | <0.001 | 0.918 | 0.566 |
| sdo_14 | 0.858 | 0.053 | 16.038 | <0.001 | 0.858 | 0.492 |
| sdo_15 | 2.235 | 0.133 | 16.780 | <0.001 | 2.235 | 0.642 |
| sdo_16 | 1.261 | 0.075 | 16.863 | <0.001 | 1.261 | 0.664 |
| Authoritarian Aggression | 0.104 | 0.031 | 3.389 | 0.001 | 0.214 | 0.214 |
| Authoritarian Submission | 0.315 | 0.047 | 6.678 | <0.001 | 0.511 | 0.511 |
| Conventionalism | 0.200 | 0.036 | 5.548 | <0.001 | 0.550 | 0.550 |
| SDO | 0.691 | 0.092 | 7.505 | <0.001 | 0.914 | 0.914 |
| RWA | 0.345 | 0.046 | 7.511 | <0.001 | 0.904 | 0.904 |

Based on Pratto et al. [1] several of the SDO items were recoded before building the scores and running the SEM; thus, all SDO items load positively on the SDO latent factor; Root Mean Square Error of Approximation = 0.070, Comparative Fit Index = 0.787, Tucker-Lewis Index = 0.768, Standardized Root Mean Square Residual = 0.067; SDO = Social Dominance Orientation, RWA = Right-Wing Authoritarianism.

## Detailed Results on the Structural Equation Model on Personal Values Types

**Supplementary Table 3. Detailed Results of the Structural Equation Model.**

**Latent variables:**

| | | Estimate | SE | z | p | Std.lv | Std.all |
|---|---|---|---|---|---|---|---|
| Self-Direction | | | | | | | |
| | pvq_1 | 1.000 | | | | 0.733 | 0.580 |
| | pvq_11 | 0.578 | 0.085 | 6.831 | <0.001 | 0.424 | 0.436 |
| Stimulation | | | | | | | |
| | pvq_6 | 1.000 | | | | 0.998 | 0.771 |
| | pvq_15 | 1.065 | 0.061 | 17.562 | <0.001 | 1.063 | 0.799 |
| Hedonism | | | | | | | |
| | pvq_10 | 1.000 | | | | 0.758 | 0.711 |
| | pvq_21 | 1.293 | 0.115 | 11.250 | <0.001 | 0.980 | 0.810 |
| Security | | | | | | | |
| | pvq_5 | 1.000 | | | | 1.023 | 0.838 |
| | pvq_14 | 0.704 | 0.073 | 9.598 | <0.001 | 0.720 | 0.544 |
| Conformity | | | | | | | |
| | pvq_7 | 1.000 | | | | 0.825 | 0.600 |
| | pvq_16 | 0.954 | 0.089 | 10.741 | <0.001 | 0.786 | 0.599 |
| Universalism | | | | | | | |
| | pvq_3 | 1.000 | | | | 0.433 | 0.427 |
| | pvq_8 | 1.031 | 0.146 | 7.048 | <0.001 | 0.446 | 0.469 |
| | pvq_19 | 1.179 | 0.155 | 7.589 | <0.001 | 0.510 | 0.554 |
| Benevolence | | | | | | | |
| | pvq_12 | 1.000 | | | | 0.843 | 0.819 |
| | pvq_18 | 0.489 | 0.048 | 10.110 | <0.001 | 0.412 | 0.497 |
| Achievement | | | | | | | |
| | pvq_4 | 1.000 | | | | 0.811 | 0.638 |
| | pvq_13 | 1.240 | 0.103 | 12.080 | <0.001 | 1.005 | 0.805 |
| Power | | | | | | | |
| | pvq_2 | 1.000 | | | | 0.839 | 0.626 |
| | pvq_17 | 0.839 | 0.072 | 11.611 | <0.001 | 0.704 | 0.591 |
| Openness to Change | | | | | | | |
| | Self-Direction | 1.000 | | | | 0.761 | 0.761 |
| | Stimulation | 1.658 | 0.174 | 9.525 | <0.001 | 0.928 | 0.928 |
| | Hedonism | 0.838 | 0.109 | 7.707 | <0.001 | 0.617 | 0.617 |
| Conservation | | | | | | | |
| | Security | 1.000 | | | | 0.783 | 0.783 |
| | Conformity | 1.060 | 0.103 | 10.256 | <0.001 | 1.029 | 1.029 |
| Self-Transcendence | | | | | | | |
| | Universalism | 1.000 | | | | 0.869 | 0.869 |
| | Benevolence | 2.055 | 0.252 | 8.153 | <0.001 | 0.917 | 0.917 |
| Self-Enhancement | | | | | | | |
| | Achievement | 1.000 | | | | 0.790 | 0.790 |
| | Power | 1.514 | 0.177 | 8.530 | <0.001 | 1.155 | 1.155 |

**Regressions:**

| | | Estimate | SE | z | p | Std.lv | Std.all |
|---|---|---|---|---|---|---|---|
| Openness to Change | | | | | | | |
| | SEEKING | 0.705 | 0.092 | 7.675 | <0.001 | 1.263 | 0.448 |
| | FEAR | -0.286 | 0.065 | -4.425 | <0.001 | -0.512 | -0.265 |
| | CARE | -0.112 | 0.058 | -1.928 | 0.054 | -0.200 | -0.089 |
| | ANGER | 0.039 | 0.043 | 0.904 | 0.366 | 0.070 | 0.034 |

| | | | | | | | |
|---|---|---|---|---|---|---|---|
| | PLAY | 0.471 | 0.076 | 6.210 | <0.001 | 0.844 | 0.339 |
| | SADNESS | 0.197 | 0.073 | 2.700 | 0.007 | 0.353 | 0.149 |
| | Age | -0.006 | 0.002 | -2.691 | 0.007 | -0.012 | -0.124 |
| | Gender (0 = male) | 0.029 | 0.048 | 0.616 | 0.538 | 0.053 | 0.025 |
| | Education (0 = no/school degree) | -0.119 | 0.051 | -2.308 | 0.021 | -0.213 | -0.100 |
| Conservation | | | | | | | |
| | SEEKING | -0.386 | 0.118 | -3.265 | 0.001 | -0.482 | -0.171 |
| | FEAR | 0.567 | 0.104 | 5.445 | <0.001 | 0.708 | 0.366 |
| | CARE | 0.298 | 0.100 | 2.977 | 0.003 | 0.372 | 0.165 |
| | PLAY | -0.152 | 0.110 | -1.377 | 0.169 | -0.190 | -0.076 |
| | SADNESS | -0.319 | 0.121 | -2.647 | 0.008 | -0.399 | -0.169 |
| | Age | -0.007 | 0.004 | -1.640 | 0.101 | -0.008 | -0.089 |
| | Gender (0 = male) | 0.084 | 0.083 | 1.019 | 0.308 | 0.105 | 0.050 |
| | Education (0 = no/school degree) | 0.014 | 0.088 | 0.159 | 0.874 | 0.017 | 0.008 |
| Self-Transcendence | | | | | | | |
| | SEEKING | 0.195 | 0.051 | 3.848 | <0.001 | 0.519 | 0.184 |
| | CARE | 0.536 | 0.070 | 7.636 | <0.001 | 1.426 | 0.631 |
| | ANGER | -0.124 | 0.034 | -3.695 | <0.001 | -0.329 | -0.161 |
| | PLAY | 0.038 | 0.042 | 0.905 | 0.366 | 0.102 | 0.041 |
| | SADNESS | 0.055 | 0.040 | 1.354 | 0.176 | 0.146 | 0.062 |
| | Age | 0.001 | 0.002 | 0.947 | 0.344 | 0.004 | 0.041 |
| | Gender (0 = male) | 0.029 | 0.033 | 0.893 | 0.372 | 0.078 | 0.037 |
| | Education (0 = no/school degree) | -0.074 | 0.035 | -2.084 | 0.037 | -0.197 | -0.092 |
| Self-Enhancement | | | | | | | |
| | SEEKING | 0.335 | 0.089 | 3.783 | <0.001 | 0.524 | 0.186 |
| | CARE | -0.218 | 0.073 | -2.997 | 0.003 | -0.340 | -0.151 |
| | ANGER | 0.380 | 0.064 | 5.896 | <0.001 | 0.593 | 0.290 |
| | PLAY | -0.017 | 0.078 | -0.216 | 0.829 | -0.026 | -0.011 |
| | Age | -0.009 | 0.003 | -3.176 | 0.001 | -0.015 | -0.157 |
| | Gender (0 male) | -0.144 | 0.062 | -2.325 | 0.020 | -0.225 | -0.108 |
| | Education (0 no/school degree) | 0.015 | 0.064 | 0.228 | 0.819 | 0.023 | 0.011 |

**Covariances:**

| | | Estimate | SE | z | p | Std.lv | Std.all |
|---|---|---|---|---|---|---|---|
| Openness to Change | | | | | | | |
| | Conservation | -0.162 | 0.026 | -6.174 | <0.001 | -0.568 | -0.568 |
| | Self-Transcendence | 0.021 | 0.008 | 2.605 | 0.009 | 0.221 | 0.221 |
| | Self-Enhancement | 0.025 | 0.014 | 1.733 | 0.083 | 0.111 | 0.111 |
| Conservation | | | | | | | |
| | Self-Transcendence | 0.029 | 0.014 | 2.152 | 0.031 | 0.167 | 0.167 |
| | Self-Enhancement | 0.149 | 0.030 | 4.976 | <0.001 | 0.356 | 0.356 |
| Self-Transcendence | | | | | | | |
| | Self-Enhancement | -0.018 | 0.010 | -1.802 | 0.072 | -0.129 | -0.129 |

**Variances:**

| | | Estimate | SE | z | p | Std.lv | Std.all |
|---|---|---|---|---|---|---|---|
| | pvq_01 | 1.061 | 0.104 | 10.204 | <0.001 | 1.061 | 0.664 |
| | pvq_11 | 0.765 | 0.052 | 14.770 | <0.001 | 0.765 | 0.810 |
| | pvq_06 | 0.678 | 0.058 | 11.778 | <0.001 | 0.678 | 0.405 |
| | pvq_15 | 0.642 | 0.061 | 10.563 | <0.001 | 0.642 | 0.362 |
| | pvq_10 | 0.563 | 0.056 | 10.112 | <0.001 | 0.563 | 0.495 |

| | | | | | | |
|---|---|---|---|---|---|---|
| pvq_21 | 0.504 | 0.081 | 6.192 | <0.001 | 0.504 | 0.344 |
| pvq_05 | 0.444 | 0.097 | 4.592 | <0.001 | 0.444 | 0.298 |
| pvq_14 | 1.237 | 0.084 | 14.721 | <0.001 | 1.237 | 0.704 |
| pvq_07 | 1.207 | 0.092 | 13.171 | <0.001 | 1.207 | 0.640 |
| pvq_16 | 1.104 | 0.084 | 13.207 | <0.001 | 1.104 | 0.641 |
| pvq_03 | 0.839 | 0.053 | 15.725 | <0.001 | 0.839 | 0.817 |
| pvq_08 | 0.707 | 0.047 | 15.149 | <0.001 | 0.707 | 0.780 |
| pvq_19 | 0.588 | 0.044 | 13.367 | <0.001 | 0.588 | 0.693 |
| pvq_12 | 0.348 | 0.061 | 5.748 | <0.001 | 0.348 | 0.329 |
| pvq_18 | 0.518 | 0.032 | 15.999 | <0.001 | 0.518 | 0.753 |
| pvq_04 | 0.958 | 0.071 | 13.459 | <0.001 | 0.958 | 0.593 |
| pvq_13 | 0.549 | 0.077 | 7.100 | <0.001 | 0.549 | 0.352 |
| pvq_02 | 1.094 | 0.084 | 12.978 | <0.001 | 1.094 | 0.608 |
| pvq_17 | 0.924 | 0.066 | 13.995 | <0.001 | 0.924 | 0.651 |
| Self-Direction | 0.226 | 0.084 | 2.676 | 0.007 | 0.421 | 0.421 |
| Stimulation | 0.139 | 0.055 | 2.513 | 0.012 | 0.140 | 0.140 |
| Hedonism | 0.356 | 0.048 | 7.396 | <0.001 | 0.619 | 0.619 |
| Security | 0.405 | 0.101 | 4.016 | <0.001 | 0.388 | 0.388 |
| Conformity | -0.040 | 0.071 | -0.569 | 0.569 | -0.059 | -0.059 |
| Universalism | 0.046 | 0.020 | 2.273 | 0.023 | 0.245 | 0.245 |
| Benevolence | 0.114 | 0.065 | 1.752 | 0.080 | 0.160 | 0.160 |
| Achievement | 0.247 | 0.052 | 4.744 | <0.001 | 0.377 | 0.377 |
| Power | -0.235 | 0.088 | -2.657 | 0.008 | -0.333 | -0.333 |
| Openness to Change | 0.155 | 0.031 | 4.934 | <0.001 | 0.499 | 0.499 |
| Conservation | 0.526 | 0.072 | 7.348 | <0.001 | 0.821 | 0.821 |
| Self-Transcendence | 0.059 | 0.015 | 3.932 | <0.001 | 0.418 | 0.418 |
| Self-Enhancement | 0.331 | 0.059 | 5.599 | <0.001 | 0.807 | 0.807 |

The negative variances of Conformity and Power are most likely due to their high correlations with Security and Achievement, respectively (see Supplementary Table 1); Root Mean Square Error of Approximation = 0.064, Comparative Fit Index = 0.811, Tucker-Lewis Index = 0.766, Standardized Root Mean Square Residual = 0.060; PVQ = Portraits Value Questionnaire.

**Extracting Factors from KSA-3, SDO, and PVQ Scales and Conducting Structural Equation Modeling**

***Method***

An Exploratory Factor Analysis (EFA) was conducted to extract latent factors from the scale scores of the KSA-3 subscales, the SDO scale, and the PVQ scales (excluding tradition). To determine the number of factors to extract, the Kaiser-Guttman criterion (number of eigenvalues > 1), the screeplot, and parallel analyses (applying the maximum likelihood estimator) were used. These analyses revealed that both a four- and a six-factorial structure could be used. Loadings of all scales on the four/six factors were inspected by running the EFA one time with four and one time with six factors. Both times, the maximum likelihood estimator and the "oblimin" rotation were applied. The latter one allows for interrelations between the factors.

Based on the loadings, it was decided to model four factors within the final structural equation model (SEM) to be predicted by the ANPS. To build the SEM, the lavaan package in R [2] was used. More specifically, the six ANPS scales were included as manifest variables in the model and links of each ANPS scale to each of the four latent factors were modeled if the respective ANPS scale significantly correlated with at least one of the scales (i.e., KSA-3 subscales, SDO scale, PVQ scales) included in the respective latent factor. The maximum likelihood estimator was used. The SEM analyses were based on men and women, only, to allow gender to be included as additional dummy-coded variable. Moreover, age and education (dummy-coded) were included in the model as manifest variables.

***Results***

*Exploratory Factor Analyses*

The Kaiser, Meyer, Olkin measure of sampling adequacy based on the correlation matrix of the three KSA-3 subscales, the SDO scale, and the nine PVQ scales was 0.72. Moreover, the Bartlett test revealed a significant score ($Chi^2 = 2282.52$, $p < 0.001$). Both results indicate that a factorization can be implemented.

Parallel analysis revealed that six latent factors should be extracted by an EFA, however, only four eigenvalues exceeding 1 were found. Additionally, a screeplot indicated one or four factors to be extracted. For reasons of transparency, loadings of all scales on the four and six factors are presented in Supplementary Table 4.

The four-factor solution was used for further analyses for the following reasons: 1. The four-factor solution did not comprise factors with only one underlying scale (as did the six-factor solution), 2. The four-factor solution fitted better to the theory since all three RWA scales loaded most strongly on the same factor and the value types loaded on factors in line with the model of Schwartz (e.g., self-direction, stimulation, and hedonism all showed their highest loadings on the same factor).

**Supplementary Table 4. Factor loadings of the KSA-3, SDO, and PVQ scales based on the four- and six-factorial EFA solutions.**

| | Factor | | | | | | | | | |
|---|---|---|---|---|---|---|---|---|---|---|
| | 1 | 2 | 3 | 4 | 1 | 2 | 3 | 4 | 5 | 6 |
| KSA-3 Authoritarian Aggression | 0.14 | 0.05 | -0.22 | **0.58** | 0.00 | **0.83** | -0.06 | 0.01 | 0.00 | -0.04 |
| KSA-3 Authoritarian Submission | 0.03 | 0.12 | -0.11 | **0.61** | 0.04 | **0.52** | 0.02 | -0.01 | 0.03 | 0.18 |
| KSA-3 Conven-tionalism | -0.12 | 0.08 | -0.28 | **0.56** | -0.06 | 0.15 | -0.22 | 0.01 | -0.04 | **0.57** |
| SDO | 0.07 | 0.18 | **-0.71** | 0.24 | -0.04 | 0.19 | **-0.64** | 0.24 | 0.09 | 0.24 |
| Self-Direction | 0.12 | **0.41** | 0.16 | -0.16 | -0.04 | 0.05 | 0.21 | **0.49** | 0.11 | -0.15 |
| Stimulation | 0.02 | **0.90** | 0.05 | -0.03 | 0.18 | 0.01 | 0.13 | **0.72** | 0.06 | 0.11 |
| Hedonism | 0.11 | **0.47** | 0.03 | -0.02 | **0.99** | 0.01 | -0.02 | 0.01 | 0.00 | -0.01 |
| Achievement | **0.62** | 0.05 | 0.08 | 0.03 | -0.01 | -0.12 | 0.03 | 0.00 | **0.81** | 0.03 |
| Power | **0.90** | 0.01 | -0.05 | 0.00 | 0.03 | 0.18 | -0.13 | 0.04 | **0.67** | -0.12 |
| Security | 0.10 | -0.18 | 0.15 | **0.66** | 0.07 | **0.40** | 0.18 | **-0.36** | 0.10 | 0.23 |
| Conformity | 0.07 | -0.33 | 0.17 | **0.58** | -0.08 | 0.24 | 0.18 | **-0.45** | 0.14 | 0.26 |
| Benevolence | -0.05 | 0.18 | **0.52** | 0.31 | 0.07 | -0.06 | **0.53** | 0.03 | 0.04 | 0.34 |
| Universalism | -0.07 | 0.20 | **0.77** | 0.05 | -0.05 | 0.02 | **0.83** | 0.18 | -0.05 | -0.01 |

Bold writing indicates the factor on which the respective scale loads most strongly; N = 626. SDO = Social Dominance Orientation, KSA-3 = Short Scale on Authoritarianism.

*Structural Equation Modeling including Latent Factors based on KSA-3, SDO, and PVQ scales*

The relations between the ANPS (manifest variables) and the four latent factors were modeled by means of an SEM. In detail, links between each ANPS scale and factors 2 and 4 were modeled, while only links between SEEKING, CARE, ANGER, PLAY and factor 1 and between SEEKING, CARE, ANGER, PLAY, SADNESS and factor 3 were modeled based on results of correlational analyses.

Running this model revealed the results as presented in Supplementary Figure 1 and Supplementary Table 5. In detail, latent factor 1 (based on the Achievement and Power scales) was positively related to SEEKING and ANGER and negatively to CARE. Latent factor 2 (based on the Self-direction, Stimulation, and Hedonism scales) was positively associated to SEEKING, PLAY, and SADNESS, and it was negatively related to FEAR. The third latent factor (based on SDO (positively) and Benevolence and Universalism (negatively)) was positively linked to ANGER and negatively to SEEKING and CARE. Finally, latent factor 4 (based on the three KSA-3 scales, and the Security and Conformity scales) was negatively related to SEEKING and positively by FEAR.

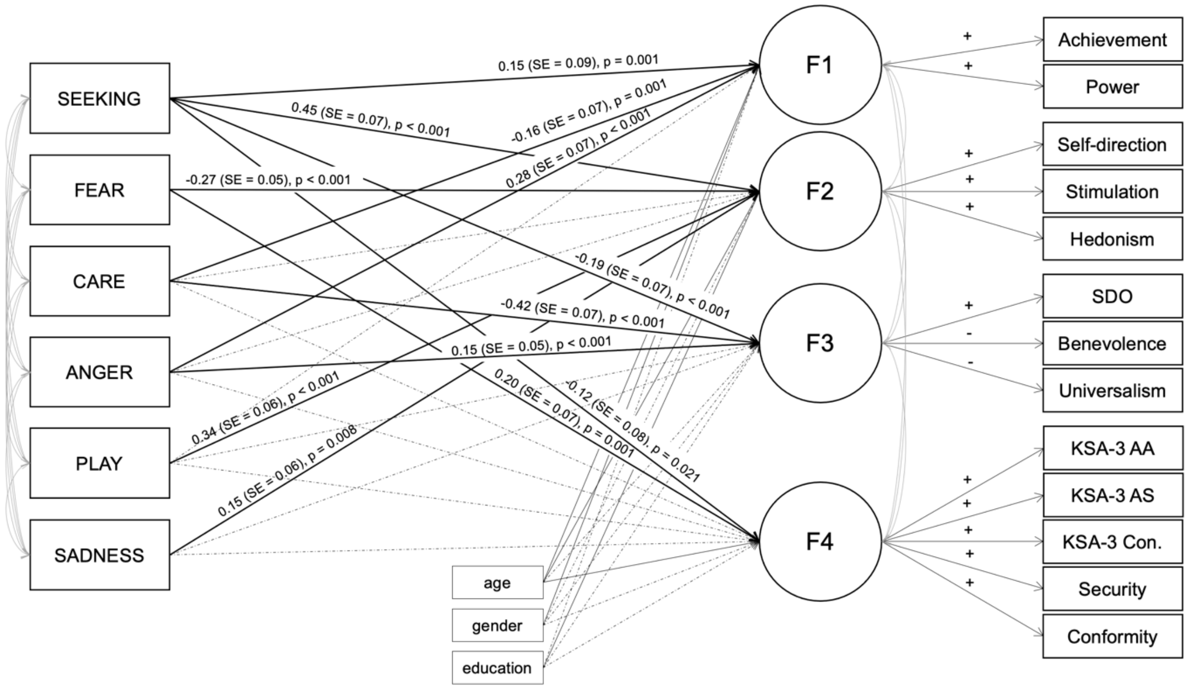


**Supplementary Figure 1. Results of the structural equation model.** Dashed lines indicate associations which were modeled but turned out to be non-significant; only estimates of significant associations between the ANPS and the latent factors are presented; all estimates are standardized estimates; variances are not included in the figure for easier interpretability; similarly, only estimates of the significant relations between the ANPS and the latent factors are presented for easier readability. SDO = Social Dominance Orientation, KSA-3 = Short Scale on Authoritarianism, AA = Authoritarian Aggression, AS = Authoritarian Submission, Con. = Conventionalism.

**Supplementary Table 5. Detailed Results of the Structural Equation Model.**

**Latent variables:**

| | | Estimate | SE | z | p | Std.lv | Std.all |
|---|---|---|---|---|---|---|---|
| F1 | | | | | | | |
| | Achievement | 1.000 | | | | 0.677 | 0.618 |
| | Power | 1.387 | 0.132 | 10.504 | <0.001 | 0.939 | 0.897 |
| F2 | | | | | | | |
| | Self-Direction | 1.000 | | | | 0.441 | 0.496 |
| | Stimulation | 2.135 | 0.200 | 10.669 | <0.001 | 0.942 | 0.799 |
| | Hedonism | 1.243 | 0.134 | 9.251 | <0.001 | 0.549 | 0.543 |
| F3 | | | | | | | |
| | SDO | 1.000 | | | | 0.551 | 0.640 |
| | Benevolence | -0.754 | 0.069 | -10.967 | <0.001 | -0.416 | -0.532 |
| | Universalism | -1.040 | 0.077 | -13.512 | <0.001 | -0.573 | -0.850 |
| F4 | | | | | | | |
| | KSA-3 AA | 1.000 | | | | 0.596 | 0.697 |
| | KSA-3 AS | 0.909 | 0.071 | 12.836 | <0.001 | 0.541 | 0.632 |
| | KSA-3 Con. | 0.631 | 0.057 | 11.062 | <0.001 | 0.376 | 0.529 |
| | Security | 1.213 | 0.091 | 13.339 | <0.001 | 0.723 | 0.665 |
| | Conformity | 1.089 | 0.090 | 12.087 | <0.001 | 0.649 | 0.586 |

**Regressions:**

| | | Estimate | SE | z | p | Std.lv | Std.all |
|---|---|---|---|---|---|---|---|
| F1 | | | | | | | |
| | SEEKING | 0.292 | 0.088 | 3.300 | 0.001 | 0.431 | 0.153 |
| | CARE | -0.242 | 0.074 | -3.258 | 0.001 | -0.358 | -0.158 |
| | ANGER | 0.380 | 0.065 | 5.858 | <0.001 | 0.562 | 0.275 |
| | PLAY | -0.004 | 0.080 | -0.056 | 0.955 | -0.007 | -0.003 |
| | Age | -0.009 | 0.003 | -3.077 | 0.002 | -0.014 | -0.146 |
| | Gender (0 = male) | -0.163 | 0.063 | -2.561 | 0.010 | -0.240 | -0.115 |
| | Education (0 = no/school degree) | 0.012 | 0.066 | 0.186 | 0.852 | 0.018 | 0.009 |
| F2 | | | | | | | |
| | SEEKING | 0.555 | 0.069 | 8.048 | <0.001 | 1.258 | 0.446 |
| | FEAR | -0.234 | 0.051 | -4.605 | <0.001 | -0.529 | -0.274 |
| | CARE | -0.086 | 0.046 | -1.879 | 0.060 | -0.196 | -0.087 |
| | ANGER | 0.049 | 0.036 | 1.355 | 0.175 | 0.112 | 0.055 |
| | PLAY | 0.378 | 0.058 | 6.469 | <0.001 | 0.856 | 0.344 |
| | SADNESS | 0.153 | 0.058 | 2.658 | 0.008 | 0.347 | 0.147 |
| | Age | -0.005 | 0.002 | -2.652 | 0.008 | -0.011 | -0.122 |
| | Gender (0 = male) | 0.021 | 0.038 | 0.548 | 0.584 | 0.047 | 0.022 |
| | Education (0 = no/school degree) | -0.093 | 0.041 | -2.271 | 0.023 | -0.210 | -0.098 |
| F3 | | | | | | | |
| | SEEKING | -0.290 | 0.071 | -4.064 | <0.001 | -0.526 | -0.186 |
| | CARE | -0.522 | 0.066 | -7.887 | <0.001 | -0.947 | -0.419 |
| | ANGER | 0.167 | 0.047 | 3.518 | <0.001 | 0.302 | 0.148 |
| | PLAY | 0.001 | 0.064 | 0.021 | 0.984 | 0.002 | 0.001 |
| | SADNESS | -0.031 | 0.059 | -0.521 | 0.602 | -0.056 | -0.024 |
| | Age | -0.001 | 0.002 | -0.495 | 0.621 | -0.002 | -0.022 |
| | Gender (0 = male) | -0.086 | 0.049 | -1.740 | 0.082 | -0.156 | -0.075 |
| | Education (0 = no/school degree) | 0.085 | 0.052 | 1.616 | 0.106 | 0.154 | 0.072 |

| | | | | | | | |
|---|---|---|---|---|---|---|---|
| F4 | | | | | | | |
| | SEEKING | -0.195 | 0.084 | -2.310 | 0.021 | -0.327 | -0.116 |
| | FEAR | 0.225 | 0.071 | 3.184 | 0.001 | 0.378 | 0.195 |
| | CARE | 0.080 | 0.071 | 1.118 | 0.264 | 0.134 | 0.059 |
| | ANGER | 0.065 | 0.057 | 1.150 | 0.250 | 0.110 | 0.054 |
| | PLAY | -0.038 | 0.079 | -0.486 | 0.627 | -0.064 | -0.026 |
| | SADNESS | -0.116 | 0.084 | -1.377 | 0.169 | -0.194 | -0.082 |
| | Age | -0.006 | 0.003 | -2.214 | 0.027 | -0.011 | -0.116 |
| | Gender (0 = male) | -0.067 | 0.060 | -1.133 | 0.257 | -0.113 | -0.054 |
| | Education (0 = no/school degree) | -0.076 | 0.063 | -1.200 | 0.230 | -0.127 | -0.060 |

**Covariances:**

| | | Estimate | SE | z | p | Std.lv | Std.all |
|---|---|---|---|---|---|---|---|
| F1 | | | | | | | |
| | F2 | 0.022 | 0.012 | 1.848 | 0.065 | 0.115 | 0.115 |
| | F3 | 0.085 | 0.018 | 4.832 | <0.001 | 0.300 | 0.300 |
| | F4 | 0.147 | 0.023 | 6.274 | <0.001 | 0.422 | 0.422 |
| F2 | | | | | | | |
| | F3 | -0.024 | 0.009 | -2.596 | 0.009 | -0.172 | -0.172 |
| | F4 | -0.037 | 0.012 | -3.214 | 0.001 | -0.211 | -0.211 |
| F3 | | | | | | | |
| | F4 | 0.084 | 0.016 | 5.324 | <0.001 | 0.323 | 0.323 |

**Variances:**

| | Estimate | SE | z | p | Std.lv | Std.all |
|---|---|---|---|---|---|---|
| Achievement | 0.741 | 0.056 | 13.155 | <0.001 | 0.741 | 0.618 |
| Power | 0.215 | 0.073 | 2.934 | 0.003 | 0.215 | 0.196 |
| Self-Direction | 0.596 | 0.037 | 16.041 | <0.001 | 0.596 | 0.754 |
| Stimulation | 0.505 | 0.061 | 8.315 | <0.001 | 0.505 | 0.362 |
| Hedonism | 0.721 | 0.046 | 15.571 | <0.001 | 0.721 | 0.706 |
| SDO | 0.437 | 0.031 | 14.179 | <0.001 | 0.437 | 0.590 |
| Benevolence | 0.438 | 0.028 | 15.852 | <0.001 | 0.438 | 0.717 |
| Universalism | 0.126 | 0.020 | 6.463 | <0.001 | 0.126 | 0.278 |
| KSA-3 AA | 0.376 | 0.029 | 13.020 | <0.001 | 0.376 | 0.514 |
| KSA-3 AS | 0.442 | 0.031 | 14.407 | <0.001 | 0.442 | 0.601 |
| KSA-3 Con. | 0.364 | 0.023 | 15.773 | <0.001 | 0.364 | 0.721 |
| Security | 0.659 | 0.048 | 13.764 | <0.001 | 0.659 | 0.558 |
| Conformity | 0.804 | 0.053 | 15.104 | <0.001 | 0.804 | 0.656 |
| F1 | 0.377 | 0.052 | 7.232 | <0.001 | 0.824 | 0.824 |
| F2 | 0.095 | 0.018 | 5.428 | <0.001 | 0.490 | 0.490 |
| F3 | 0.211 | 0.027 | 7.756 | <0.001 | 0.695 | 0.695 |
| F4 | 0.322 | 0.037 | 8.664 | <0.001 | 0.908 | 0.908 |

Root Mean Square Error of Approximation = 0.103, Comparative Fit Index = 0.699, Tucker-Lewis Index = 0.589, Standardized Root Mean Square Residual = 0.076. SDO = Social Dominance Orientation, KSA-3 = Short Scale on Authoritarianism, AA = Authoritarian Aggression, AS = Authoritarian Submission, Con. = Conventionalism.

**References**


1. Pratto F, Sidanius J, Stallworth LM, Malle BF (1994) Social dominance orientation: A personality variable predicting social and political attitudes. Journal of Personality and Social Psychology 67:741–763

2. Rosseel Y (2012) lavaan: an {R} package for structural equation modeling; version 0.6-8. Journal of Statistical Software 48:1–36
